# Iron(III)-Catalyzed Synthesis of 2-Alkyl Homoallyl Sulfonyl Amides: Antiproliferative Study and Reactivity Scope of Aza-Prins Cyclization

**DOI:** 10.1021/acs.joc.2c01267

**Published:** 2022-08-03

**Authors:** Rubén M. Carballo, José M. Padrón, Israel Fernández, Daniel A. Cruz, Luana Grmuša, Víctor S. Martín, Juan I. Padrón

**Affiliations:** † Laboratorio de Química Farmacéutica, Facultad de Química, Universidad Autónoma de Yucatán, Calle 43 S/N entre calle 96 y calle 40, Col. Inalámbrica, 97069 Mérida, Yucatán, México; ‡ Instituto Universitario de Bio-Orgánica “Antonio González”, Universidad de La Laguna, C/Francisco Sánchez 2, 38206 La Laguna, Spain; § Departamento de Química Orgánica I and Centro de Innovación en Química Avanzada (ORFEO-CINQA), Facultad de Ciencias Químicas, Universidad Complutense de Madrid, Quimica Organica, Av. Complutense, 28040 Madrid, Spain; ∥ Instituto de Productos Naturales y Agrobiología, Consejo Superior de Investigaciones Científicas (IPNA-CSIC), Avda. Astrofísico Francisco Sánchez 3, 38206 La Laguna, Tenerife Islas Canarias, Spain

## Abstract

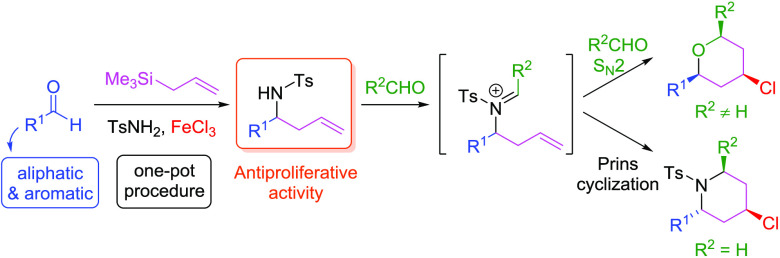

A direct, catalytic, and complementary method to obtain 2-substituted homoallyl sulfonyl amides is described, starting from sulfonyl amides, aldehydes, and allyltrimethylsilane using iron(III) chloride as a sustainable catalyst. The scope of the process and the reactivity in aza-Prins cyclization is evaluated and supported by density functional theory (DFT) studies. Finally, an evaluation of the antiproliferative activity for this family of sulfonyl amides is also included.

## Introduction

Organic compounds including nitrogen in their structures are widely distributed in nature and exhibit a vast range of interesting biological activities, with the six-membered heterocycles standing out among them.^1−5^ More than 85% of biologically active compounds are heterocycles, often forming part of complex molecules as a structural backbone.^6^ On the other hand, a significant number of the 200 most sold drugs are derivatives of aliphatic amines that also serve as a substructure in a wide variety of agrochemicals, textiles, and other materials.^7−10^

**Figure 1:**
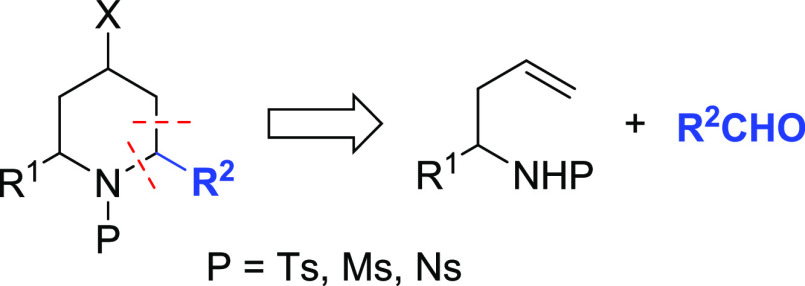
2-Substituted homoallyl sulfonyl amides as precursors of 4-halo-2,6-disubstituted piperidine in aza-Prins annulation.

Aza-Prins cyclization is a powerful synthetic tool that couples an unsaturated activated amine to a carbonyl reactant, building three new bonds through the process and permitting access to medium-size azacycles present in both natural and synthetic compounds.^11,12^ In two previous reports, we described the direct aza-Prins cyclization between homoallyl sulfonyl amides and aldehydes, using iron(III) salts as catalysts to provide 4-halo monosubstituted six-membered ring azacycles.^13^ The method uses homoallyl sulfonyl amides, taking into account the similar chemical reactivity of the nitrogen of sulfonyl amides to that of the hydroxyl in their oxygenated counterparts.^14^ Now, our initial intention is to synthesize 4-halo-2,6-disubstituted piperidine from 2-substituted homoallyl sulfonyl amides and aldehydes catalyzed by iron(III) salts (Figure 1).

Homoallylic sulfonyl amides are powerful synthons used as key intermediates in the preparation of complex molecules and in total syntheses. A quick search through the literature will find various methods to prepare these molecules from diverse source materials, using several catalysts.^15−20^ In 2015, Fan et al. reported an FeCl_3_-catalyzed three-component reaction between aldehydes, sulfonamides, and allylsilanes that provides a way to construct 2-substituted homoallyl sulfonyl amide derivatives.^21^ However, this methodology is incompatible with aliphatic aldehydes. Therefore, in the present article, we report a smoother approach compatible with both aromatic and aliphatic aldehydes, as well as its application toward the aza-Prins annulation. A computational study of the reaction mechanism is included. Moreover, the antiproliferative activity of the readily synthesized homoallyl sulfonamides is also discussed; to the best of our knowledge, this has not been considered previously.

## Results and Discussion

**Scheme 1:**
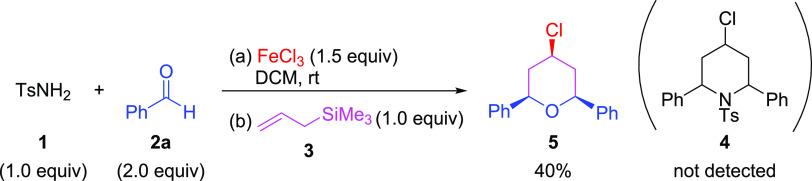
Approach to 4-Chloro-2,6-disubstituted Piperidine

Considering that FeCl_3_ is known to catalyze the reaction between tosylamide (**1**), benzaldehyde (**2a**), and allyltrimethylsilane (**3**) to form 2-substituted homoallyl sulfonyl amides,^21^ we ponder exploring the direct synthesis of 4-halo-2,6-disubstituted piperidines by adding 2 equiv of benzaldehyde and 1.5 equiv of FeCl_3_ (Scheme 1). In these reaction conditions, the formation of the desired piperidine (**4**) was not detected, but 4-chloro-2,6-diphenyltetrahydro-*2H*-pyran (**5**) was obtained instead. The latter species derives from the corresponding homoallyl alcohol formed upon a reaction between benzaldehyde (**2a**) and allyltrimethylsilane (**3**) in the presence of FeCl_3_, which reacts with more benzaldehyde (**2a**) and FeCl_3_ to yield tetrahydropyran **5** through a Prins cyclization reaction.^22^

**Scheme 2:**
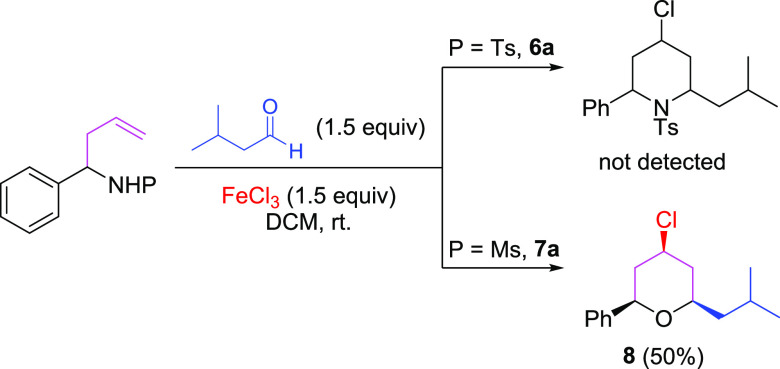
Anomalous Results in the Approach to 4-Chloro-2,6-disubstituted Piperidine

After this result, it was clear that the formation of a 2-substituted homoallyl alcohol is favored over the corresponding 2-substituted homoallyl tosylamide, so we tested aza-Prins cyclization using a premade homoallyl sulfonyl amide. The required 2-substituted homoallyl sulfonyl amides were synthesized following the procedure reported by Fan et al.^21^ The *N*-(1-phenylbut-3-en-1-yl)-*p*-toluenesulfonamide (**6a**) was obtained in an 86% yield and *N*-(1-phenylbut-3-en-1-yl)methanesulfonamide (**7a**) in a 69% yield. Compounds **6a** and **7a** were then treated under the conditions of the aza-Prins cyclization previously reported by our research group (Scheme 2).^13^ Again, the desired piperidine was not produced in either case, whereas to our surprise, pyran 8 was obtained when the reaction was carried out with **7a**.^22^

**Scheme 3:**
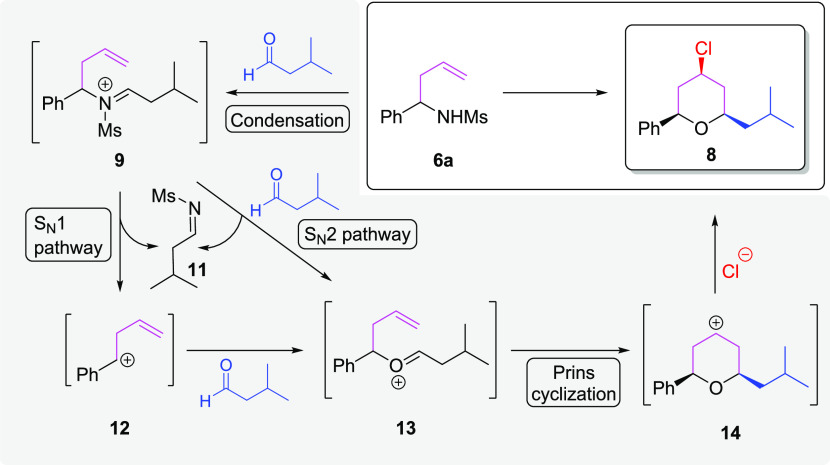
Proposed Mechanism of the Frustrated Aza-Prins Cyclization with α-Substituted Homoallyl Sulfonyl amides

This result encouraged us to propose a tentative reaction mechanism to accede to 8 (Scheme 3). Based on our previous report on the Prins reactions involving homoallyl alcohols and aldehydes,^23^ the formation of imonium ion 9 would constitute the first step of the transformation. This species then loses imine 11 to afford the carbocation 12 in an S_N_1-type reaction. Subsequently, the isovaleraldehyde would react with 12 to form oxonium ion 13, which leads through a Prins cyclization to the formation of pyran 8. Alternatively, oxonium ion 13 could also be formed directly from 9 and the aldehyde via an S_N_2 reaction.

**Figure 2:**
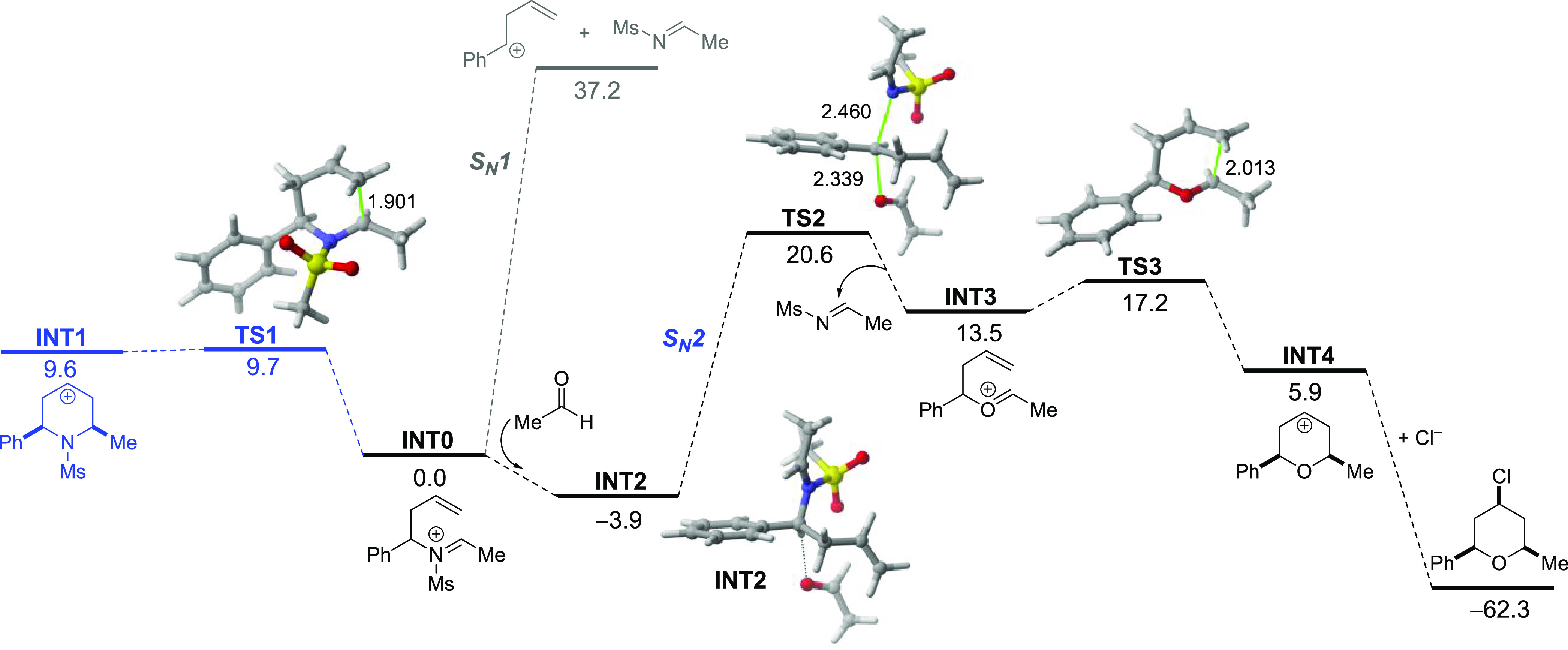
Computed reaction profile for the formation of pyrans. Relative electronic energies (ZPVE included) and bond distances are given in kcal/mol and angstroms, respectively. All data have been computed at the PCM(CH_2_Cl_2_)-M06-2X/def2-SVP level.

Density functional theory (DFT) calculations (PCM(CH_2_Cl_2_)-M06-2X/def-SVP level) were carried out to gain more insight into the mechanism involved in the above transformation. The computed reaction profile for the process involving sulfonamide 7a and acetaldehyde is shown in Figure 2, as a model of the isovaleraldehyde used experimentally.

Our calculations indicate that the initially formed imonium cation INT0 (analogous to 9 in Scheme 3) can indeed undergo an aza-Prins reaction via TS1, with a low barrier of only 9.7 kcal/mol. However, this reaction is highly endothermic (Δ*E*_R_ = 9.6 kcal/mol), which renders the reverse reaction highly feasible. Therefore, INT0 could undergo the proposed S_N_1-type reaction, leading to carbocation 12 and the corresponding imine (analogous to 11 in Scheme 3). However, our calculations indicate that this fragmentation can be ruled out in view of the prohibitive computed reaction energy of 37.2 kcal/mol. Alternatively, INT0 can be transformed upon reaction with the aldehyde into intermediate INT2. This process is exothermic due to the stabilization of the positive charge of the initial iminium cation by the lone pair of the carbonyl group in the aldehyde. From INT2 onward, the proposed S_N_2-type reaction takes place through TS2 with a barrier of 20.6 kcal/mol, feasible at room temperature. The readily formed oxonium cation INT3 undergoes the expected cyclization reaction, ending with a C–Cl bond formation. The highly exothermic nature of the last step is likely promoted by FeCl_4_^–^.^23^ It compensates for the endothermicity of the previous steps and drives the process forward toward the formation of the experimentally observed pyran (8 in Scheme 3).

**Scheme 4:**
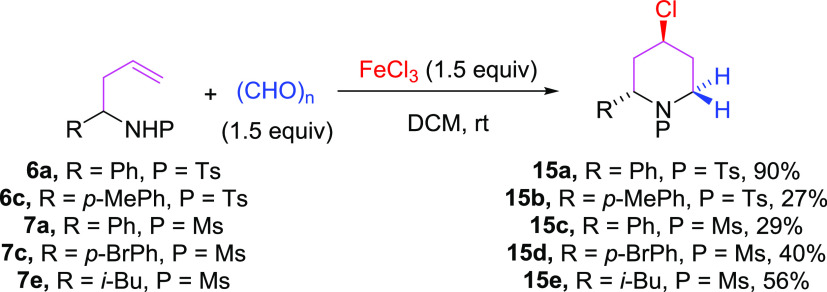
Aza-Prins Cyclization with Formaldehyde from 2-Substituted Homoallyl Sulfonyl Amides

Aza-Prins cyclization was attempted with other aldehydes (benzaldehyde, 2-phenylacetaldehyde, octanal, cyclohexanecarboxaldehyde) and other sources of iron(III) (Fe(acac)_3_/TMSCl), but unfortunately, the desired 4-chloro-2,6-disubstituted piperidines were not detected to be obtained instead the corresponding tetrahydropyrans. However, when the reaction was carried out using formaldehyde, we obtained the expected 4-chloro-2-disubstituted piperidine 15 in a 90% yield (Scheme 4).^24^ Other a-substituted homoallyl sulfonamides react with formaldehyde to give 4-chloro-2-substituted piperidines (15a–e) in moderate yields (Scheme 4). These results show that steric hindrances are also involved in the course of the reaction

To investigate the influence of this moiety on the 2-substituted homoallyl sulfonyl amides in aza-Prins cyclization, 2-alkyl homoallyl sulfonyl amides were required. Since the methodology reported by Fan et al. was incompatible with aliphatic aldehydes, we developed a modified version to also obtain 2-alkyl homoallyl sulfonyl amides.

Initially, we ran the imine formation process in situ using 1.5 equiv of tosylamide (**1**), 1.0 equiv of benzaldehyde (**2a**) and 5 mol % of FeCl_3_ in dry CH_2_Cl_2_ (0.1 M) for 3 h at room temperature. After this time, 1.0 equiv of allyltrimethylsilane (**3**) and an extra 5 mol % of iron(III) chloride were added. The homoallyl sulfonyl amide **6a** was obtained in a 60% yield (Table 1, entry 1). Up to this point, we had assumed that adding an extra 5 mol % FeCl_3_ was crucial to increase performance (60% yield vs 53% by Fan et al.). These authors reported a concentration of 0.2 M in their procedure, so we decided to increase it in our reaction (from 0.1 to 0.3 M), improving the yield up to 65% (Table 1, entry 2). Then, in search of milder reaction conditions than those reported before, a fine-tuning of the reaction by adjusting temperature and the amount of Lewis acid and addition of a desiccant agent such as magnesium sulfate allowed us to increase the yield of **6a** to an excellent 90% (Table 1, entries 3–7). The reaction temperature in this one-pot process is not only helpful to increase the yield but also to improve the reaction rate.

**Table 1:**
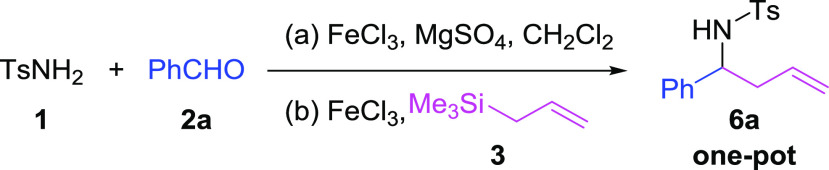
Optimization of the Iron-Catalyzed One-Pot Synthesis of 2-Substituted Homoallyl Sulfonyl Amides

entry^a^	*X*	FeX_3_ (mol %)	MgSO_4_ (equiv)	TsNH_2_ (equiv)	TMSCl (equiv)	temperature (°C)	time^d^ (h)	yield^b^ (%)
1	Cl	5 (a), 5 (b)		1.5		rt (a), rt (b)	7	60^c^
2	Cl	5 (a), 5 (b)		1.5		rt (a), rt (b)	7	65
3	Cl	5 (a), 5 (b)	1.0	1.5		50 (a), rt (b)	7	70
4	Cl	5 (a), 5 (b)	1.0	1.5		50 (a), 0 (b)	7	75
5	Cl	5 (a), 10 (b)	1.0	1.5		50 (a), 0 (b)	6	80
6	Cl	10 (a), 5 (b)	1.0	1.5		50 (a), 0 (b)	6	82
7	Cl	10 (a), 10 (b)	1.0	1.5		50 (a), 0 (b)	5	90
8	Cl	10 (a), 10 (b)		1.5	1.0 (a)	50 (a), 0 (b)	5	80
9	Cl	10 (a), 10 (b)		1.5	1.0 (b)	50 (a), 0 (b)	5	78
10	Cl	10 (a), 10 (b)		1.5	1.0 (a, b)	50 (a), 0 (b)	5	83
11	acac	10 (a), 10 (b)		1.5		50 (a), 0 (b)	24	0
12	acac	10 (a), 10 (b)		1.5	1.0 (b)	50 (a), 0 (b)	24	0
13	acac	10 (a), 10 (b)		1.5	1.0 (a)	50 (a), 0 (b)	12	30
14	acac	10 (a), 10 (b)		1.5	1.0 (a, b)	50 (a), 0 (b)	12	60

a Conditions: **1** (4.2 mmol), **2** (2.8 mmol), FeCl_3_ (10 mol %), CH_2_Cl_2_ (0.3 M), MgSO_4_ (4.2 mmol), or TMSCl (4.2 mmol), reflux during 2 h, then FeCl_3_ (10 mol %), allyl trimethyl silane (2.8 mmol) at 0 °C.

b Isolated yields.

c 0.1 M in CH_2_Cl_2_.

d Includes 0.5 h for step **a**.

Therefore, we settled on the use of 10 mol % of FeCl_3_ for each reaction, 1.0 equiv of MgSO_4_, 1.5 equiv of sulfonyl amide **1**, and 1.0 equiv of benzaldehyde (**2a**). Addition of TMSCl instead of MgSO_4_ to activate the tosylimine in the allyl addition afforded similar or slightly lower yields (Table 1, entries 8–10). Other iron(III) sources such as Fe(acac)_3_ only catalyzed the one-pot process in combination with TMSCl, but with lower yields and longer reaction times (Table 1, entries 11–14).

Next, we investigated the scope of the process with a variety of aldehydes. We tested aliphatic and aromatic aldehydes under the optimized reaction conditions, using several sulfonyl amides (tosyl (**1**), mesyl (**16**), and nosylamides (**17**)). In general, the corresponding homoallyl sulfonyl amides (**6a–l** and **7a–e**) were obtained in good yields (Table 2). This one-pot procedure works well with a wide range of aromatic and aliphatic aldehydes, except when isovaleraldehyde was used (Table 2, entries 12 and 17). Isovaleraldehyde is not substituted at α so its probability to enolize is higher than the rest of the example. This could be the reason why the yield is so low. Unfortunately, when the reaction was carried out with sulfonyl amides (**16** and **17**, mesyl, and nosyl) and benzaldehydes with electron-withdrawing groups, the desired compounds (**7f**, **7g**, **15a**, and **15b**) were not obtained (Table 2, entries 18–21). The reactivity of the substituted benzaldehydes follows the described net electrophilicity (*E*) values to a significant extent, which is a more refined way to determine the electron-accepting or -donating character of the molecules.^25^

**Table 2:**
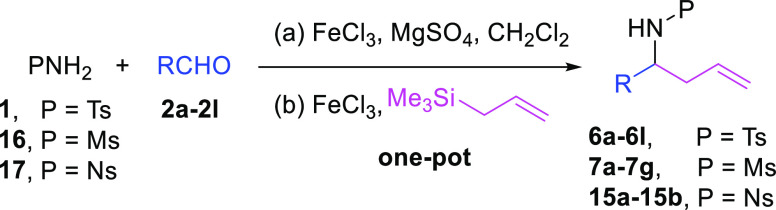
Scope of the One-Pot Synthesis of 2-Substituted Homoallyl Sulfonyl Amides

entry^a^	*R*	*P*	product	yield (%)^b^	yield reported (%)
1	Ph	Ts	**6a**	90	94 (86)^c^
2	1-Naph	Ts	**6b**	85	81
3	*p*-MePh	Ts	**6c**	65	93
4	*p*-FPh	Ts	**6d**	75	73
5	*p*-ClPh	Ts	**6e**	85	79
6	*o*-ClPh	Ts	**6f**	87	
7	*p*-BrPh	Ts	**6g**	87	
8	*p*-NO_2_Ph	Ts	**6h**	60	
9	*p*-MeO_2_CPh	Ts	**6i**	78	
10	*c*-Hex	Ts	**6j**	67	
11	*t*-Bu	Ts	**6k**	80	
12	*i*-Bu	Ts	**6l**	16	
13	Ph	Ms	**7a**	77	72 (70)
14	*p*-MePh	Ms	**7b**	60	
15	*p*-BrPh	Ms	**7c**	75	
16	*c*-Hex	Ms	**7d**	69	
17	*i*-Bu	Ms	**7e**	20	
18	*p*-NO_2_Ph	Ms	**7f**	0	
19	*p*-MeO_2_CPh	Ms	**7g**	0	
20	Ph	Ns	**15a**	0	
21	Naph	Ns	**15b**	0	

a Conditions: **1** (4.2 mmol), **2** (2.8 mmol), FeCl_3_ (10 mol %), CH_2_Cl_2_ (0.3 M), MgSO_4_ (4.2 mmol), or TMSCl (4.2 mmol), reflux during 2 h, then FeCl_3_ (10 mol %), allyltrimethyl silane (2.8 mmol) at 0 °C.

b Isolated yields.

c (In parentheses, the yields obtained in our hands).

Thus, the highest yield was for the benzaldehyde derivative with the highest *E*, the *p*-bromobenzaldeyde (Table 2, entries 7 and 15), and the lowest was for the one with the lowest E value, *p*-methylbenzaldehyde (Table 2, entries 3 and 14). Between both extremes, we noted that substitutions with *p*-fluoro and *p*-chloro have a very good correlation with E values (Table 2, entries 4 and 5). In addition, the yields obtained for *p*- and *o*-chlorobenzaldehyde were almost identical, showing that steric effects have no influence during this one-pot reaction (Table 2, entries 5 and 6). In the benzaldehyde derivatives with groups able to interact with iron(III) salts, such as NO_2_ and CO_2_Me, the yields were lower (Table 2, entries 8 and 9). In obtaining aliphatic sulfonamides, the yields span from moderate to good except for isovaleraldehyde (Table 2, entries 10, 11, 12, 16, and 17). In general, tosylamine led to better yields than mesylamide, while nosylamide showed no reaction at all (Table 2, entries 20 and 21). This behavior is based on the varying nucleophilicity of the sulfonyl amides.

Compared with the yields reported by Fan et al., those obtained with our methodology are better (Table 2, entries 1, 2, 4, 5, and 13), except when *p*-methylbenzaldehyde was used (Table 2, entry 3). Furthermore, our methodology is compatible with aliphatic aldehydes.

With 2-alkyl homoallyl sulfonyl amides in hand, aza-Prins cyclization was tested but unfortunately, the desired piperidines were not obtained either except with formaldehyde where a variety of 4-chloro-2-substituted piperidines were obtained (Scheme 4). At this point, we wondered about the biological properties of the 2-substituted homoallyl sulfonyl amides, discovering that there was no information regarding their in vitro bioactivity. Thus, we decided to shed light on this question by testing them against the human solid tumor cell lines A2780, HBL-100, HeLa, SW1573, T-47D, and WiDr (Table 3, Supporting information).

**Scheme 5:**
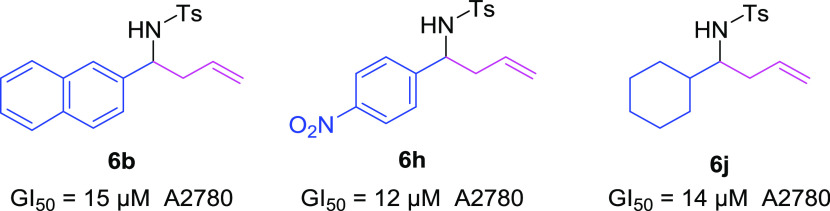
2-Substituted Homoallyl Tosylamides with the Best in Vitro Antiproliferative Activity against the Human Solid Tumor Cell Lines A2780, HBL-100, HeLa, SW1573, T-47D, and WiDr

During the biological testing stage, lipophilicity and *in vitro* antiproliferative activity were measured. The amine-protecting group provided the first quote regarding the SAR since all of the active compounds bear the tosyl group (Table 3, Supporting information), **6b, 6h**, and **6j**, showing the best antiproliferative performance (Scheme 5). Measurements of lipophilicity ranged from 3.81 to 5.24 for active compounds, which is not a significant difference. No correlation was found between bioactivity profiles and lipophilicity values.

## Conclusions

We have developed a procedure using FeCl_3_ as a sustainable catalyst to obtain 2-alkyl homoallyl sulfonyl amides complementing the methodology reported by Fan et al. This procedure is also compatible with using aromatic aldehydes to obtain 2-aromatic homoallyl sulfonyl amides. In general, better yields are obtained than those reported previously. Unfortunately, the 2-substituted homoallyl sulfonyl amides do not work as starting substrates for aza-Prins cyclization, leading to 4-halo-2,6-disubstituted tetrahydropyrans. According to DFT calculations, this is due to a more favorable alternative reaction pathway that involves an S_N_2-type reaction, leading to the formation of an oxonium cation intermediate that produces pyrans instead. The involvement of steric factors cannot be ruled out as well, as evidenced by the fact that with formaldehyde the reaction works.

In addition, the antiproliferative activity of this type of compound is reported for the first time, showing a moderate antiproliferative activity against six cancer cell lines. Compound **6h** was the most active, showing a GI_50_ = 12 μM against cell line A2780.

## Experimental Section

### General Remarks

All reagents were obtained from commercial sources and used without further purification. Solvents were dried and distilled before use. Column chromatography was performed using a silica gel (0.015–0.04 mm) and *n*-hexane/EtOAc solvent systems. For analytical thin-layer chromatography, silica gel-ready foils were used, being developed with 254 nm UV light and/or sprayed with a solution of ninhydrin (10% w/v in EtOH) or vanillin in EtOH:H_2_SO_4_:AcOH (15:1:1.3) and heating at 200 °C. The ^1^H NMR spectra were recorded at 300 MHz, while ^13^C NMR spectra were recorded at 75 MHz, VTU 298.0 K. Chemical shifts were reported in parts per million. The residual solvent peak was used as an internal reference. IR spectra were recorded on a Bruker IFS 55 spectrometer model. Elemental analyses were performed using an EA 1108 CHNS-O FISONS instrument.

### General Procedure for the Synthesis of 2-Substituted Homoallyl Sulfonyl Amides

To a solution of TsNH_2_ or MsNH_2_ (4.2 mmol, 1.5 equiv) in dry CH_2_Cl_2_ (0.3 M), MgSO_4_ (4.2 mmol, 1.5 equiv) and aldehyde (2.8 mmol, 1.0 equiv) were added. After 5 min, FeCl_3_ (0.28 mmol, 0.1 equiv) was added and the mixture was heated for 2 h under reflux (heat-on block system). Upon completion, it was allowed to warm to room temperature and then cooled to 0 °C. Allyltrimethylsilane (2.8 mmol, 1.0 equiv) and FeCl_3_ (0.28 mmol, 0.1 equiv) were added. The reaction was stirred until TLC analysis showed the complete formation of the product. The reaction was then quenched by addition of water and extracted with CH_2_Cl_2_. Combined organic layers were dried over MgSO_4_, and the solvent was removed under reduced pressure. This crude reaction mixture was purified by flash silica gel column chromatography and automated flash silica gel chromatography (*n*-hexane/EtOAc/DCM solvent systems).

### Characterization Data of Compounds in Table 2 and Scheme 4

For compounds **6a–l**, **7a**, **15a–c**, and **15e**, the spectroscopic data coincide with those reported in the literature.^13,21,26−32^ Compounds **7b–e** and **15d** were fully characterized.

#### *N*-(1-*p*-Tolylbut-3-enyl)methanesulfonamide (**7b**)

Flash column chromatography eluent system (*n*-hexane/EtOAc/DCM 19:1:20), yield 60% (403 mg), as an amorphous solid. ^1^H NMR (CDCl_3_, 400 MHz): δ 7.22 (m, 4H), 5.71 (m, 1H), 5.17 (m, 3H), 4.53 (quint, *J* = 7.1 Hz, 1H), 2.57 (m, 5H), 2.36 (s, 3H). 13C{1H} NMR (100 MHz, CDCl_3_): δ 137.9 (C), 137.7 (C), 133.4 (CH), 129.5 (2CH), 126.7 (2CH), 119.1 (CH2), 57.2 (CH), 42.0 (CH2), 41.9 (CH3), 21.1 (CH3). FTIR (cm^–1^): 3278.0, 2939.9, 1647.8, 1314.8, 1154.8. Anal. calcd for C_12_H_17_NO_2_S: C, 60.22; H, 7.16; N, 5.85. Found: C, 60.34; H, 7.18; N, 5.90.

#### *N*-(1-(4-Bromophenyl)but-3-enyl)methanesulfonamide (**7c**)

Flash column chromatography eluent system (*n*-hexane/EtOAc/DCM 19:1:20), yield 75% (639 mg), as an amorphous solid. ^1^H NMR (CDCl_3_, 300 MHz): δ 7.46 (d, *J* = 7.6 Hz, 2H), 7.20 (d, *J* = 7.6 Hz, 2H), 5.63 (m, 2H), 5.08 (m, 2H), 4.47 (quint, *J* = 7.2 Hz, 1H), 2.60 (m, 3H), 2.50 (t, *J* = 6.7 Hz, 2H). 13C{1H} NMR (75 MHz, CDCl_3_): δ 140.0 (C), 132.7 (CH), 131.7 (2CH), 128.3 (2CH), 121.5 (C), 119.2 (CH2), 56.8 (CH), 41.7 (CH3), 41.5 (CH2). FTIR (cm^–1^): 3273.4, 2929.5, 1641.5, 1314.4, 1151.8. Anal. calcd for C_11_H_14_BrNO_2_S: C, 43.43; H, 4.64; N, 4.60. Found: C, 43.44; H, 4.63; N, 4.61.

#### *N*-(1-Cyclohexylbut-3-enyl)methanesulfonamide (**7d**)

Flash column chromatography eluent system (*n*-hexane/EtOAc/DCM 19:1:20), yield 69% (447 mg), as an amorphous solid. ^1^H NMR (CDCl_3_, 400 MHz): δ 5.73 (m, 1H), 5.06 (m, 2H), 4.93 (d, *J* = 8.9 Hz, 1H), 3.17 (brs, 1H), 2.88 (s, 3H), 2.23 (m, 2H), 1.65 (m, 5H), 1.39 (brs, 1H), 1.08 (m, 5H). 13C{1H} NMR (100 MHz, CDCl_3_): δ 134.2 (CH), 118.1 (CH2), 58.4 (CH), 41.7 (CH), 41.2 (CH3), 36.8 (CH2), 29.1 (CH2), 28.0 (CH2), 26.0 (CH2), 25.9 (CH2), 25.9 (CH2). FTIR (cm^–1^): 3286.8, 2928.2, 2854.1, 1641.6, 1313.9, 1152.2. Anal. calcd for C_11_H_21_NO_2_S: C, 57.11; H, 9.15; N, 6.05. Found: C, 57.12; H, 9.00; N, 6.24.

#### *N*-(6-Methylhept-1-en-4-yl)methanesulfonamide (**7e**)

Flash column chromatography eluent system (*n*-hexane/EtOAc/DCM 19:1:20), yield 20% (115 mg), as an amorphous solid. ^1^H NMR (CDCl_3_, 400 MHz): δ 5.76 (m, 1H), 5.12 (m, 2H), 4.40 (d, *J* = 7.8 Hz, 1H), 3.50 (m, 1H), 2.95 (s, 3H), 2.26 (m, 2H), 1.71 (m, 1H), 1.32 (m, 2H), 0.92 (s, 3H), 0.90 (s, 3H). 13C{1H} NMR (100 MHz, CDCl_3_): δ 133.2 (CH), 118.8 (CH2), 51.7 (CH), 44.3 (CH2), 41.9 (CH3), 40.1 (CH2), 24.3 (CH), 22.6 (CH3), 21.9 (CH3). FTIR (cm^–1^): 3289.0, 2927.9, 2854.9, 1645.9, 1314.9, 1152.6. Anal. calcd for C_9_H_19_NO_2_S: C, 52.65; H, 9.33; N, 6.82. Found: C, 53.00; H, 9.43; N, 6.90.

#### *Trans*-2-(4-Bromophenyl)-4-chloro-1-(methylsulfonyl)piperidine (**15d**)

Automated flash chromatography eluent system (*n*-hexane/EtOAc from 93:7 to 40:60), yield 40% (46 mg), as an amorphous solid. ^1^H NMR (CDCl_3_, 400 MHz): δ 7.52 (d, *J* = 8.5 Hz, 2H), 7.25 (d, *J* = 8.5 Hz, 2H), 5.26 (brs, 1H), 4.03–3.84 (m, 2H), 3.05 (ddd, *J* = 2.7, 13.0 and 15.3, 1H), 2.98 (s, 3H), 2.82 (d, *J* = 13.9 Hz, 1H), 2.26-2.16 (ddd, *J* = 5.5, 13.6 & 14.2 Hz, 1H), 2.16-2.07 (d, *J* = 12.9 Hz, 1H), 1.88 (ddd, *J* = 4.7, 12.9 and 25.0 Hz, 1H). 13C{1H} NMR (100 MHz, CDCl_3_): δ 136.4 (C), 132.2 (2 × CH), 128.3 (2 × CH), 121.7 (C), 55.6 (CH), 52.3 (CH), 41.3 (CH2), 41.1 (CH3), 38.6 (CH2), 35.9 (CH2). FTIR (cm^–1^): 2960.7, 1488.8, 1456.2, 1323.9, 1142.2. HRMS (APCI+): *m*/*z* calcd for C_12_H_16_NO_2_S^35^Cl^79^Br: 351.9774 [M + H]^+^; found: 351.9770.

## Supporting Information Available

^1^H NMR and 13C{1H} NMR spectra of all synthesized homoallyl sulfonyl amides and piperidines (Figures S1–S46) and computational details and Cartesian coordinates for all species (PDF)

## References

[ref1] Chen Q.-B.; Gao J.; Zou G.-A.; Xin X.-L.; Aisa H. A. Piperidine Alkaloids with Diverse Skeletons from Anacyclus Pyrethrum. J. Nat. Prod. 2018, 81, 1474–1482. 10.1021/acs.jnatprod.8b00239. 29775308

[ref2] Mlostoń G.; Kowalczyk M.; Celeda M.; Gach-Janczak K.; Janecka A.; Jasiński M. Synthesis and Cytotoxic Activity of Lepidilines A–D: Comparison with Some 4,5-Diphenyl Analogues and Related Imidazole-2-Thiones. J. Nat. Prod. 2021, 84, 3071–3079. 10.1021/acs.jnatprod.1c00797. 34808062 PMC8713287

[ref3] Okolotowicz K. J.; Dwyer M.; Ryan D.; Cheng J.; Cashman E. A.; Moore S.; Mercola M.; Cashman J. R. Novel Tertiary Sulfonamides as Potent Anti-Cancer Agents. Bioorg. Med. Chem. 2018, 26, 4441–4451. 10.1016/j.bmc.2018.07.042. 30075999

[ref4] Ding X.; Stasi L. P.; Dai X.; Long K.; Peng C.; Zhao B.; Wang H.; Sun C.; Hu H.; Wan Z.; Jandu K. S.; Philps O. J.; Chen Y.; Wang L.; Liu Q.; Edge C.; Li Y.; Dong K.; Guan X.; Tattersall F. D.; Reith A. D.; Ren F. 5-Substituted-N-Pyridazinylbenzamides as Potent and Selective LRRK2 Inhibitors: Improved Brain Unbound Fraction Enables Efficacy. Bioorg. Med. Chem. Lett. 2019, 29, 212–215. 10.1016/j.bmcl.2018.11.054. 30522952

[ref5] Fedorowicz J.; Sączewski J.; Konopacka A.; Waleron K.; Lejnowski D.; Ciura K.; Tomašič T.; Skok Ž.; Savijoki K.; Morawska M.; Gilbert-Girard S.; Fallarero A. Synthesis and Biological Evaluation of Hybrid Quinolone-Based Quaternary Ammonium Antibacterial Agents. Eur. J. Med. Chem. 2019, 179, 576–590. 10.1016/j.ejmech.2019.06.071. 31279292

[ref6] Heravi M. M.; Zadsirjan V. Prescribed Drugs Containing Nitrogen Heterocycles: An Overview. RSC Adv. 2020, 10, 44247–44311. 10.1039/D0RA09198G. 35557843 PMC9092475

[ref7] Du Y.-D.; Chen B.-H.; Shu W. Direct Access to Primary Amines from Alkenes by Selective Metal-Free Hydroamination. Angew. Chem., Int. Ed. 2021, 60, 9875–9880. 10.1002/anie.202016679. 33539628

[ref8] Trowbridge A.; Walton S. M.; Gaunt M. J. New Strategies for the Transition-Metal Catalyzed Synthesis of Aliphatic Amines. Chem. Rev. 2020, 120, 2613–2692. 10.1021/acs.chemrev.9b00462. 32064858

[ref9] Cabré A.; Verdaguer X.; Riera A. Recent Advances in the Enantioselective Synthesis of Chiral Amines via Transition Metal-Catalyzed Asymmetric Hydrogenation. Chem. Rev. 2022, 122, 269–339. 10.1021/acs.chemrev.1c00496. 34677059 PMC9998038

[ref10] Murugesan K.; Senthamarai T.; Chandrashekhar V. G.; Natte K.; Kamer P. C. J.; Beller M.; Jagadeesh R. V. Catalytic Reductive Aminations Using Molecular Hydrogen for Synthesis of Different Kinds of Amines. Chem. Soc. Rev. 2020, 49, 6273–6328. 10.1039/C9CS00286C. 32729851

[ref11] Subba Reddy B. V.; Nair P. N.; Antony A.; Lalli C.; Grée R. The Aza-Prins Reaction in the Synthesis of Natural Products and Analogues. Eur. J. Org. Chem. 2017, 2017, 1805–1819. 10.1002/ejoc.201601411.

[ref12] Abdul-Rashed S.; Holt C.; Frontier A. J. Alkynyl Prins and Alkynyl Aza-Prins Annulations: Scope and Synthetic Applications. Synthesis 2020, 52, 1991–2007. 10.1055/s-0039-1690869.

[ref13] Carballo R. M.; Ramírez M. A.; Rodríguez M. L.; Martín V. S.; Padrón J. I. Iron(III)-Promoted Aza-Prins-Cyclization: Direct Synthesis of Six-Membered Azacycles. Org. Lett. 2006, 8, 3837–3840. 10.1021/ol061448t. 16898830

[ref14] Miranda P. O.; Carballo R. M.; Martín V. S.; Padrón J. I. A New Catalytic Prins Cyclization Leading to Oxa- and Azacycles. Org. Lett. 2009, 11, 357–360. 10.1021/ol802593u. 19093859

[ref15] Masuyama Y.; Tosa J.; Kurusu Y. Imine Allylation by Allylic Trimethylsilanes via in Situ Formation of N-Tosyliminium Species from Carbonyl Compounds and Toluene-p-Sulfonamide with SnCl2 and N-Chlorosuccinimide: Regioselection and Diastereoselection. Chem. Commun. 1999, 12, 1075–1076. 10.1039/a902789k.

[ref16] Pramanik S.; Ghorai P. Re2O7-Catalyzed Three-Component Synthesis of Protected Secondary and Tertiary Homoallylic Amines. Chem. Commun. 2012, 48, 1820–1822. 10.1039/c2cc15472b. 22218363

[ref17] Yus M.; González-Gómez J. C.; Foubelo F. Diastereoselective Allylation of Carbonyl Compounds and Imines: Application to the Synthesis of Natural Products. Chem. Rev. 2013, 113, 5595–5698. 10.1021/cr400008h. 23540914

[ref18] Rani Kalita H.; Phukan P. Three-Component Synthesis of Homoallylic Carbamates. Synth. Commun. 2005, 35, 475–481. 10.1081/SCC-200048980.

[ref19] Pasunooti K. K.; Leow M. L.; Vedachalam S.; Gorityala B. K.; Liu X.-W. A General and Mild Copper-Catalyzed Three-Component Synthesis of Protected Homoallyl Amines. Tetrahedron Lett. 2009, 50, 2979–2981. 10.1016/j.tetlet.2009.04.008.

[ref20] Davis F. A.; Song M.; Augustine A. Asymmetric Synthesis of Trans-2,5-Disubstituted Pyrrolidines from Enantiopure Homoallylic Amines. Synthesis of Pyrrolidine (−)-197B. J. Org. Chem. 2006, 71, 2779–2786. 10.1021/jo052566h. 16555832 PMC2536609

[ref21] Fan X.; Zhu H.-B.; Lv H.; Guo K.; Guan Y.-H.; Cui X.-M.; An B.; Pu Y.-L. Assembly of Homoallylamine Derivatives through Iron-Catalyzed Three-Component Sulfonamidoallylation Reaction. Appl. Organomet. Chem. 2015, 29, 588–592. 10.1002/aoc.3334.

[ref22] Compounds 5 and 8 were previously synthesized by our research group Chem. - Eur. J., 2015; Vol. 21, pp 15211–15217.26471437

[ref23] Pérez S. J.; Purino M.; Miranda P. O.; Martín V. S.; Fernández I.; Padrón J. I. Prins Cyclization Catalyzed by a FeIII/Trimethylsilyl Halide System: The Oxocarbenium Ion Pathway versus the [2+2] Cycloaddition. Chem. - Eur. J. 2015, 21, 15211–15217. 10.1002/chem.201502488. 26471437

[ref24] Compound 15 was previously synthesized by our research group Org. Lett., 2006; Vol. 8 (17), , pp 3837–3840.16898830

[ref25] Pratihar S. Electrophilicity and Nucleophilicity of Commonly Used Aldehydes. Org. Biomol. Chem. 2014, 12, 5781–5788. 10.1039/C4OB00555D. 24979574

[ref26] Solin N.; Wallner O. A.; Szabó K. J. Palladium Pincer-Complex Catalyzed Allylation of Tosylimines by Potassium Trifluoro(Allyl)Borates. Org. Lett. 2005, 7, 689–691. 10.1021/ol0475010. 15704926

[ref27] Li S.-W.; Batey R. A. Allylation and Highly Diastereoselective Syn or Anti Crotylation of N-Toluenesulfonylimines Using Potassium Allyl- and Crotyltrifluoroborates. Chem. Commun. 2004, 12, 1382–1383. 10.1039/b403759f. 15179475

[ref28] Wang X.; Li J.; Zhang Y. Substitution of the Benzotriazolyl Group in N -(α-Amidoalkyl)Benzotriazoles and N -(α-Sulfonamidoalkyl)Benzotriazoles with Allylsamarium Bromide. Synth. Commun. 2003, 33, 3575–3581. 10.1081/SCC-120024744.

[ref29] Ghosh D.; Bera P. K.; Kumar M.; Abdi S. H. R.; Khan N. H.; Kureshy R. I.; Bajaj H. C. Asymmetric Allylation of Sulfonyl Imines Catalyzed by in Situ Generated Cu(Ii) Complexes of Chiral Amino Alcohol Based Schiff Bases. RSC Adv. 2014, 4, 56424–56433. 10.1039/C4RA10929E.

[ref30] Thirupathi P.; Kim S. S. Indium Triflate-Catalyzed Allylation Reactions of N-Sulfonyl Aldimines or N-Alkoxycarbonylamino p-Tolylsulfones with Allyltrimethylsilane: Synthesis of Protected Homoallylic Amines. Tetrahedron 2010, 66, 8623–8628. 10.1016/j.tet.2010.09.038.

[ref31] Carballo R. M.; Valdomir G.; Purino M.; Martín V. S.; Padrón J. I. Broadening the Synthetic Scope of the Iron(III)-Catalyzed Aza-Prins Cyclization. Eur. J. Org. Chem. 2010, 2010, 2304–2313. 10.1002/ejoc.200901372.

[ref32] Hasegawa E.; Hiroi N.; Osawa C.; Tayama E.; Iwamoto H. Application of Biphasic Reaction Procedure Using Ferric Chloride Dissolved in an Imidazolium Salt and Benzotrifluoride (FeIm-BTF Procedure) to Aza-Prins Cyclization Reaction. Tetrahedron Lett. 2010, 51, 6535–6538. 10.1016/j.tetlet.2010.10.018.

