# Supplementary material for: Iron(III)-Catalyzed Synthesis of 2-Alkyl Homoallyl Sulfonyl Amides: Antiproliferative Study and Reactivity Scope of Aza-Prins Cyclization
Source: J Org Chem. 2022 Aug 3;87(16):11000–6. doi: 10.1021/acs.joc.2c01267 (PMC9396664; doi:10.1021/acs.joc.2c01267)
Supplement: Supplementary file 1 — jo2c01267_si_001.pdf [file jo2c01267_si_001.pdf]

Supporting Information

Iron(III)-catalyzed synthesis of 2-alkyl homoallyl sulfonylamides. Antiproliferative study and reactivity scope of aza-Prins cyclization.

Rubén M. Carballo,[1] José M. Padrón,[2] Israel Fernández,[3] Daniel A. Cruz,[4] Luana Grmuša,[4] Víctor S. Martín[2] and Juan I. Padrón [4]*

[1] Laboratorio de Química Farmacéutica, Facultad de Química, Universidad Autónoma de Yucatán, Calle 43 S/N entre calle 96 y calle 40, Col. Inalámbrica, 97069 Mérida, Yucatán, México.

[2] Instituto Universitario de Bio-Orgánica "Antonio González", Universidad de La Laguna C/ Francisco Sánchez 2, 38206 La Laguna, Spain.

[3] Departamento de Química Orgánica I and Centro de Innovación en Química Avanzada (ORFEO-CINQA), Facultad de Ciencias Químicas, Universidad Complutense de Madrid, Spain.

[4] Instituto de Productos Naturales y Agrobiología, Consejo Superior de Investigaciones Científicas (IPNA-CSIC), Avda. Astrofísico Francisco Sánchez 3, 38206 La Laguna, Tenerife, Islas Canarias, Spain.

***** Correspondence: mail to: jipadron@ipna.csic.es

Nomenclature note:

The term "homoallyl sulfonylamide" is used throughout the text to describe the corresponding *N*-(1-radical-3-enyl)sulfonylamide.

Supporting index

Figure S1. ^{1}H NMR spectrum of compound 6a S4
Figure S2. ^{13}C NMR spectrum of compound 6a S4
Figure S3. ^{1}H NMR spectrum of compound 6b S5
Figure S4. ^{13}C NMR spectrum of compound 6b S5
Figure S5. ^{1}H NMR spectrum of compound 6c S6
Figure S6. ^{13}C NMR spectrum of compound 6c S6
Figure S7. ^{1}H NMR spectrum of compound 6d S7
Figure S8. ^{13}C NMR spectrum of compound 6d S7
Figure S9. ^{1}H NMR spectrum of compound 6e S8
Figure S10. ^{13}C NMR spectrum of compound 6e S8
Figure S11. ^{1}H NMR spectrum of compound 6f S9
Figure S12. ^{13}C NMR spectrum of compound 6f S9
Figure S13. ^{1}H NMR spectrum of compound 6g S10
Figure S14. ^{13}C NMR spectrum of compound 6g S10
Figure S15. ^{1}H NMR spectrum of compound 6h S11
Figure S16. ^{13}C NMR spectrum of compound 6h S11
Figure S17. ^{1}H NMR spectrum of compound 6i S12
Figure S18. ^{13}C NMR spectrum of compound 6i S12
Figure S19. ^{1}H NMR spectrum of compound 6j S13
Figure S20. ^{13}C NMR spectrum of compound 6j S13
Figure S21. ^{1}H NMR spectrum of compound 6k S14
Figure S22. ^{13}C NMR spectrum of compound 6k S14
Figure S23. ^{1}H NMR spectrum of compound 6l S15
Figure S24. ^{13}C NMR spectrum of compound 6l S15
Figure S25. ^{1}H NMR spectrum of compound 7a S16
Figure S26. ^{13}C NMR spectrum of compound 7a S16
Figure S27. ^{1}H NMR spectrum of compound 7b S17
Figure S28. ^{13}C NMR spectrum of compound 7b S17
Figure S29. ^{1}H NMR spectrum of compound 7c S18
Figure S30. ^{13}C NMR spectrum of compound 7c S18
Figure S31. ^{1}H NMR spectrum of compound 7d S19
Figure S32. ^{13}C NMR spectrum of compound 7d S19
Figure S33. ^{1}H NMR spectrum of compound 7e S20
Figure S34. ^{13}C NMR spectrum of compound 7e S20
Figure S35. ^{1}H NMR spectrum of compound 15a S21
Figure S36. ^{13}C NMR spectrum of compound 15a S21

Figure S37. ^{1}H NMR spectrum of compound 15b S22
Figure S38. ^{13}C NMR spectrum of compound 15b S22
Figure S39. ^{1}H NMR spectrum of compound 15c S23
Figure S40. ^{13}C NMR spectrum of compound 15c S23
Figure S41. ^{1}H NMR spectrum of compound 15d S24
Figure S42. ^{13}C NMR spectrum of compound 15d S24
Figure S43. ^{1}H NMR spectrum of compound 15e S25
Figure S44. ^{13}C NMR spectrum of compound 15e S25
Lipophilicity and *in vitro* antiproliferative data S26
Computational details S28
Cartesian coordinates S29

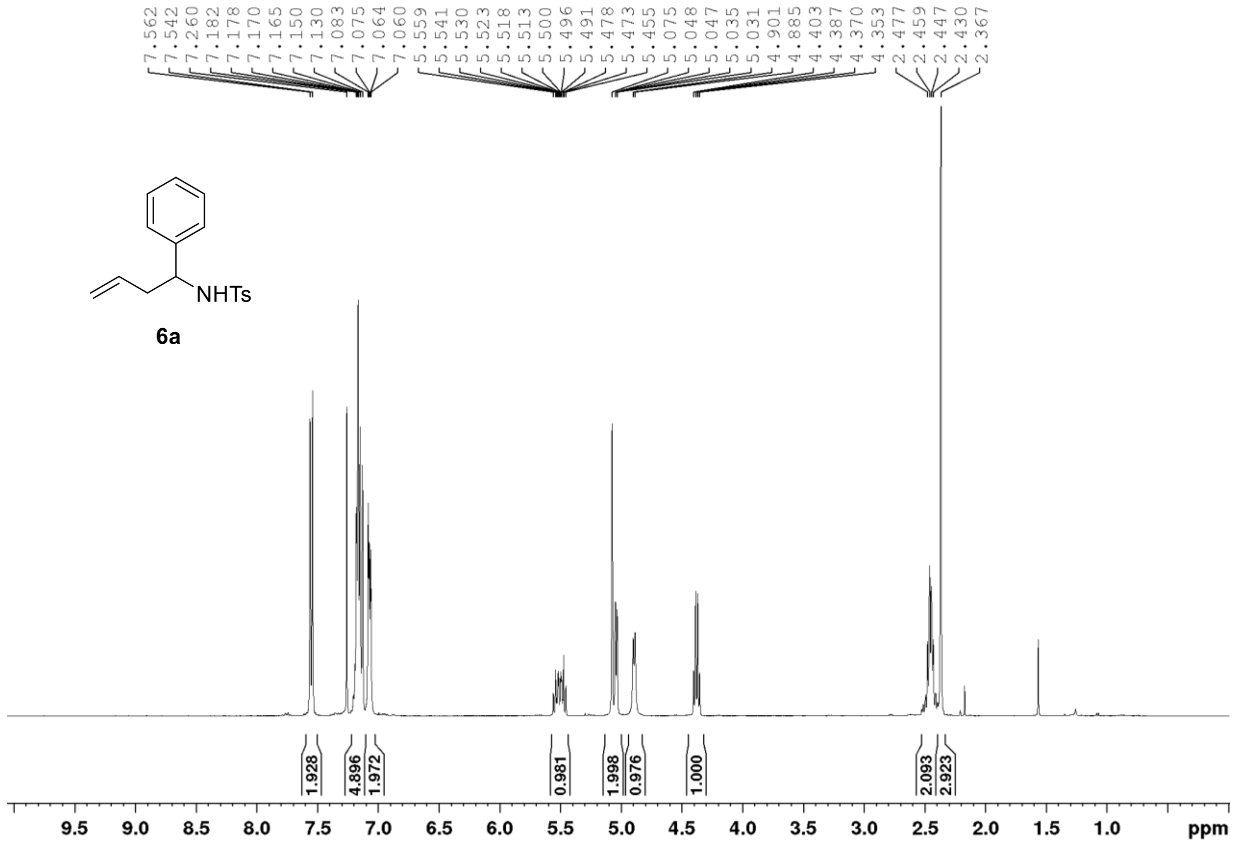

Figure S1. ^{1}H NMR ($CDCl_3$, 400 MHz) spectrum of compound **6a**

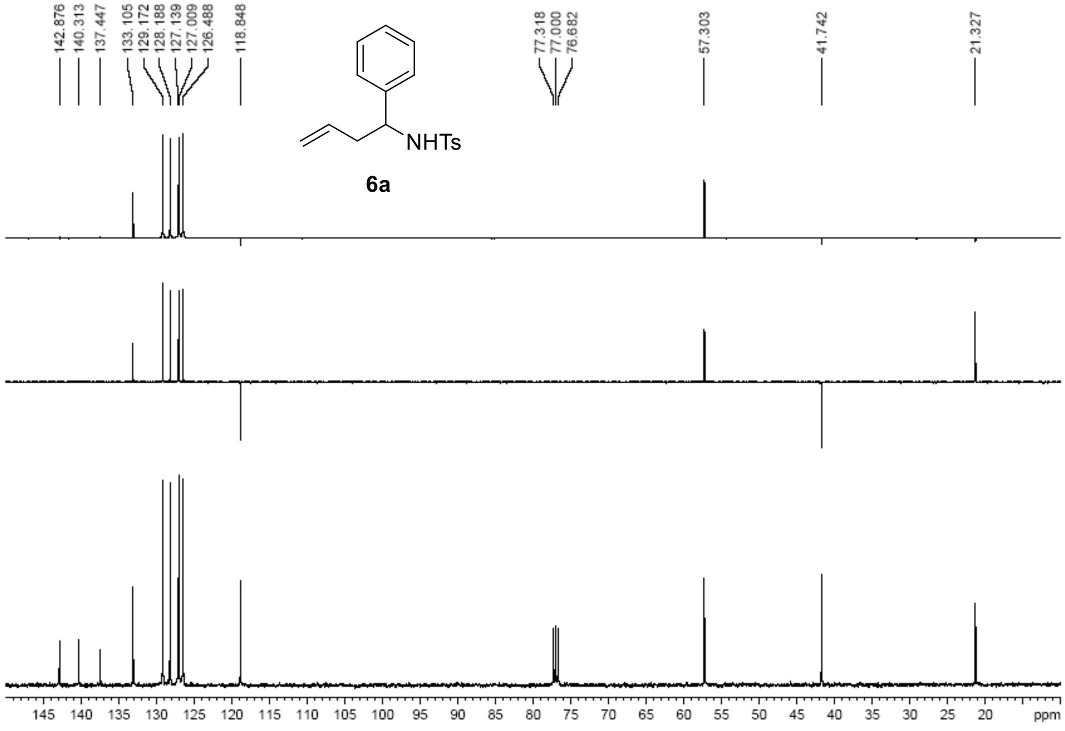

Figure S2. 13C{1H} ($CDCl_3$, 75 MHz) spectrum of compound **6a**

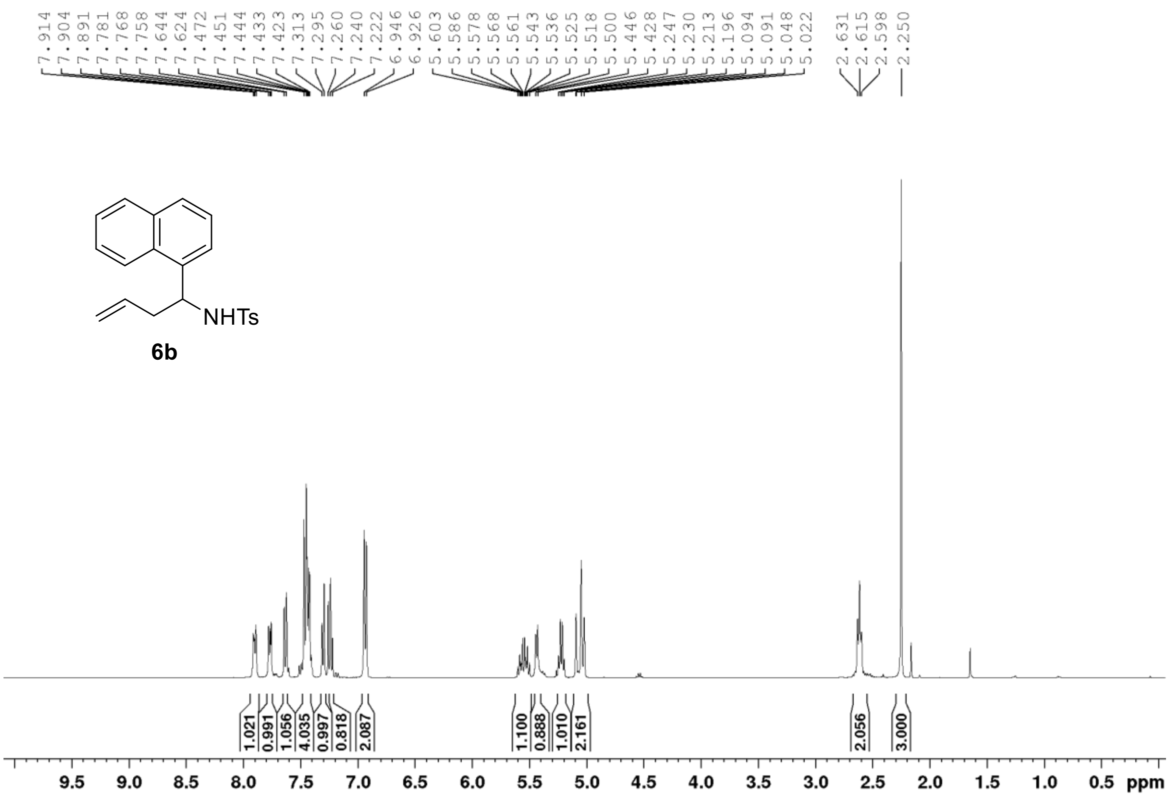

Figure S3. ^{1}H NMR ($CDCl_3$, 400 MHz) spectrum of compound **6b**

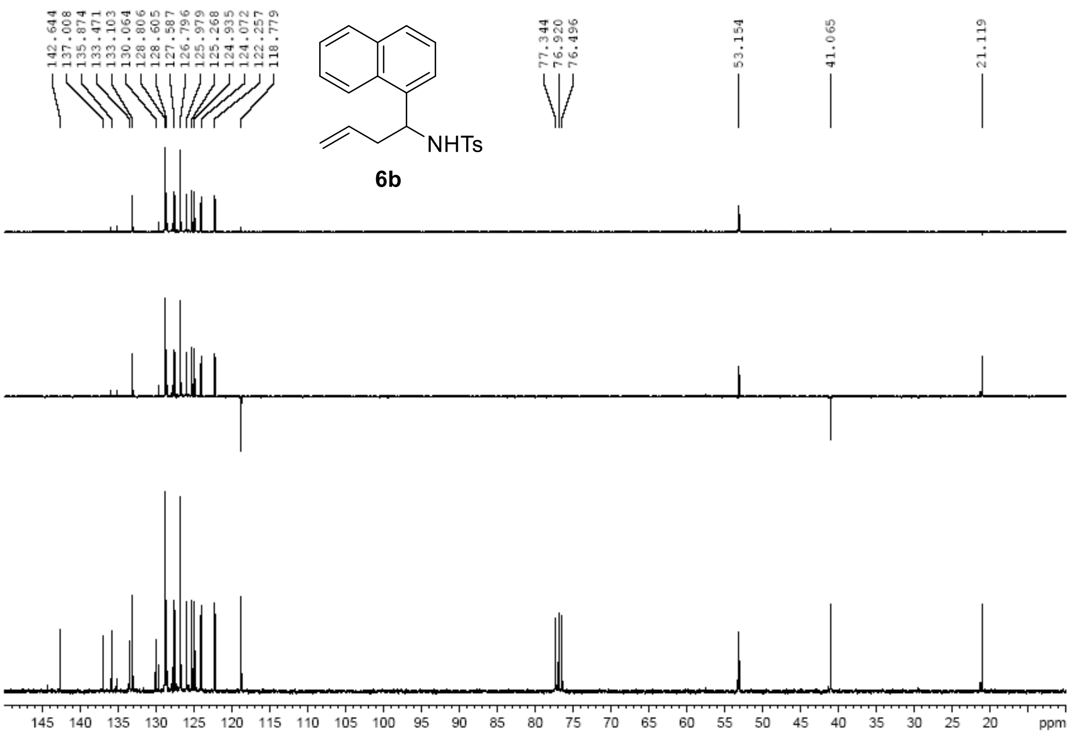

Figure S4. 13C{1H} ($CDCl_3$, 75 MHz) spectrum of compound **6b**

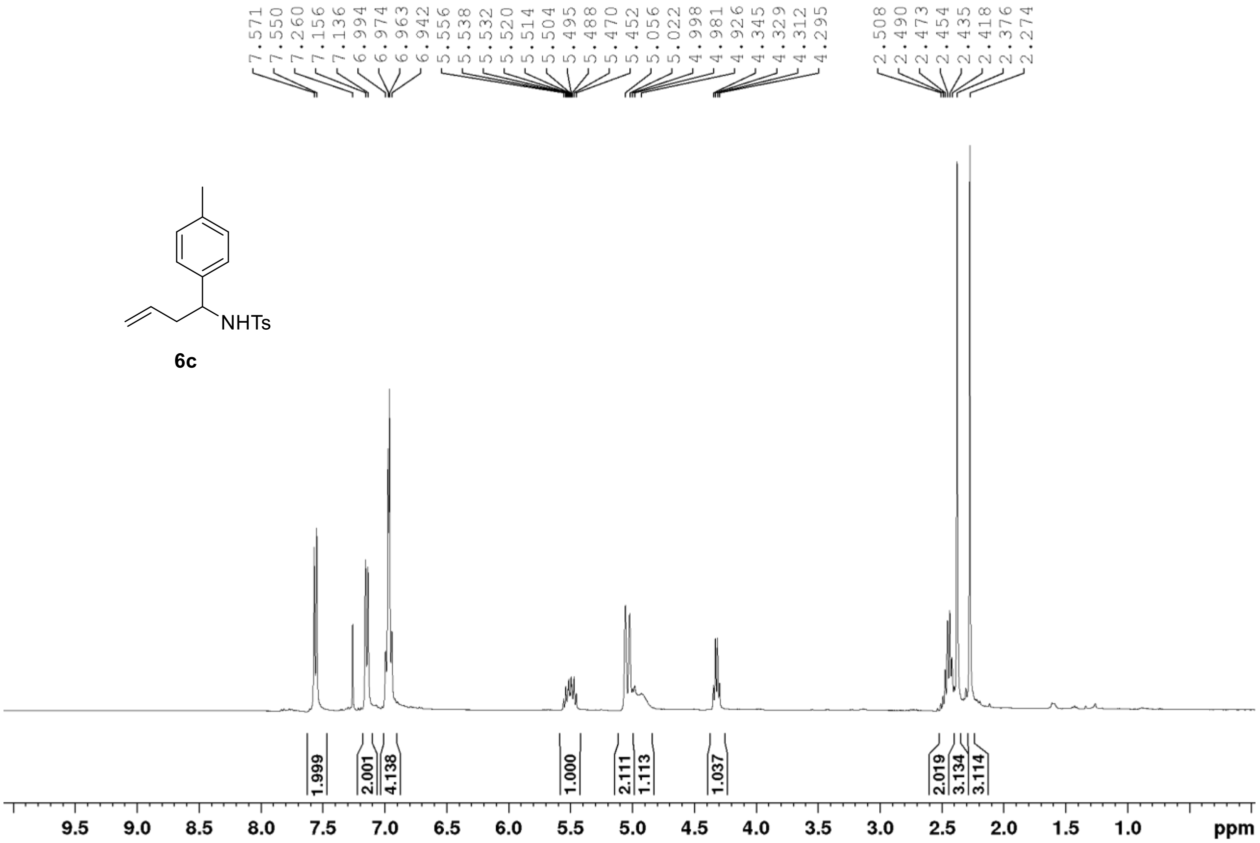

Figure S5. ^{1}H NMR ($CDCl_3$, 400 MHz) spectrum of compound **6c**

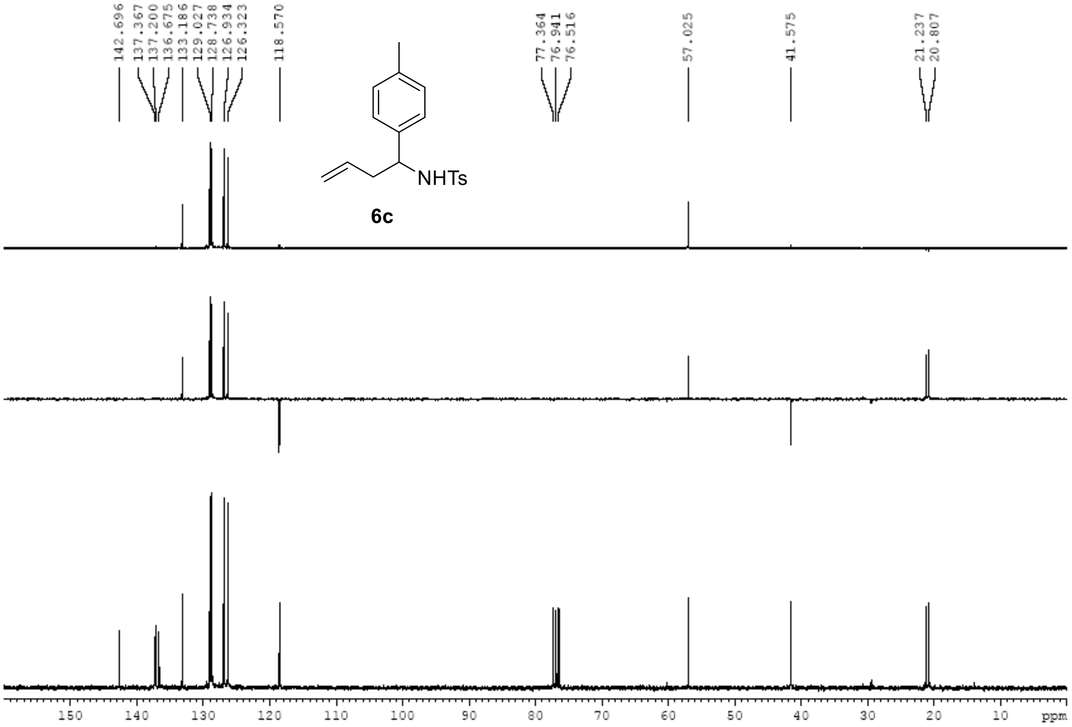

Figure S6. 13C{1H} ($CDCl_3$, 75 MHz) spectrum of compound **6c**

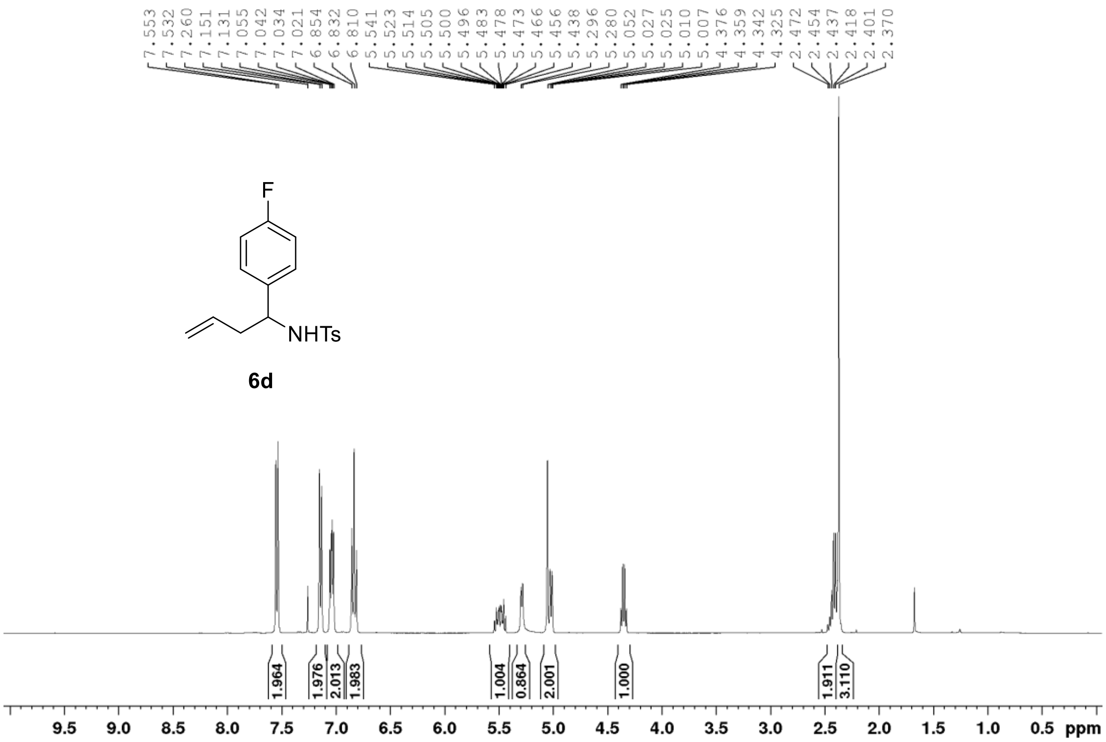

Figure S7. ^{1}H NMR ($CDCl_3$, 400 MHz) spectrum of compound **6d**

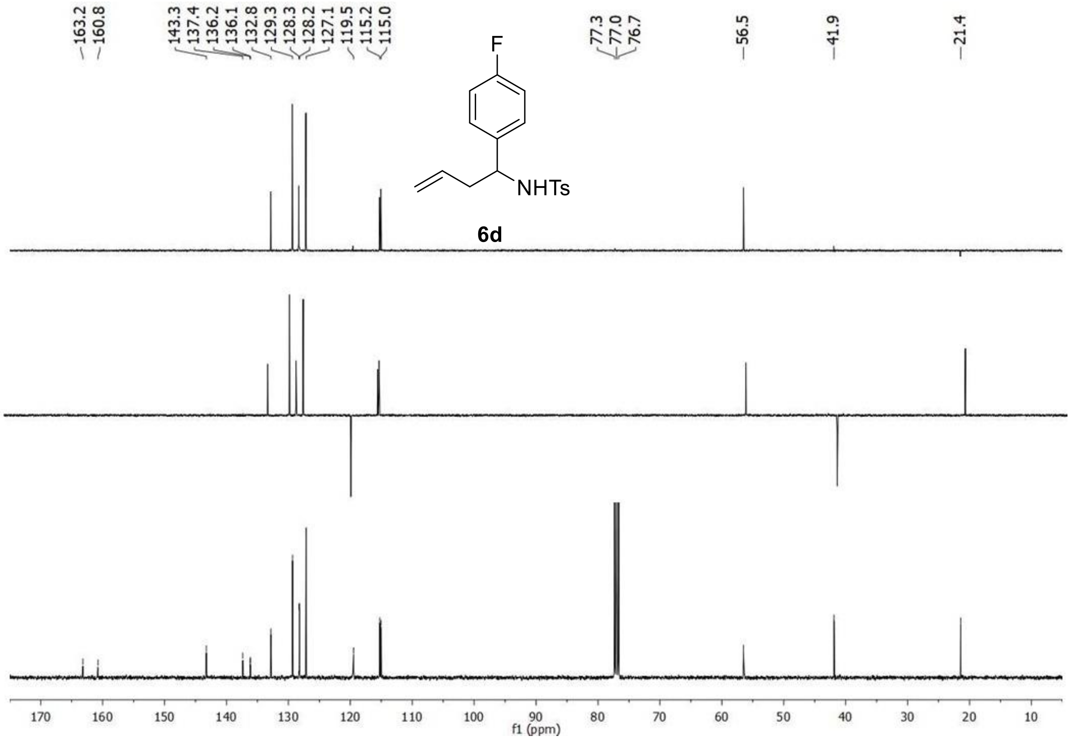

Figure S8. 13C{1H} ($CDCl_3$, 75 MHz) spectrum of compound **6d**

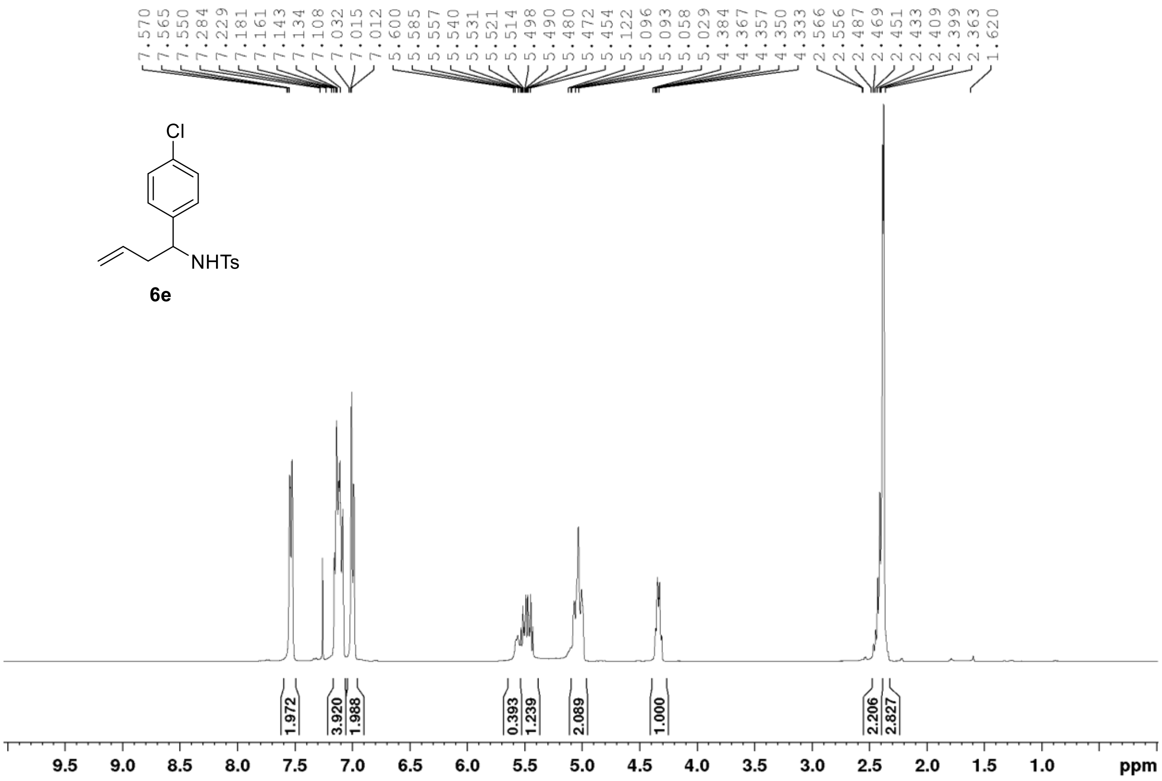

Figure S9. ^{1}H NMR ($CDCl_3$, 400 MHz) spectrum of compound **6e**

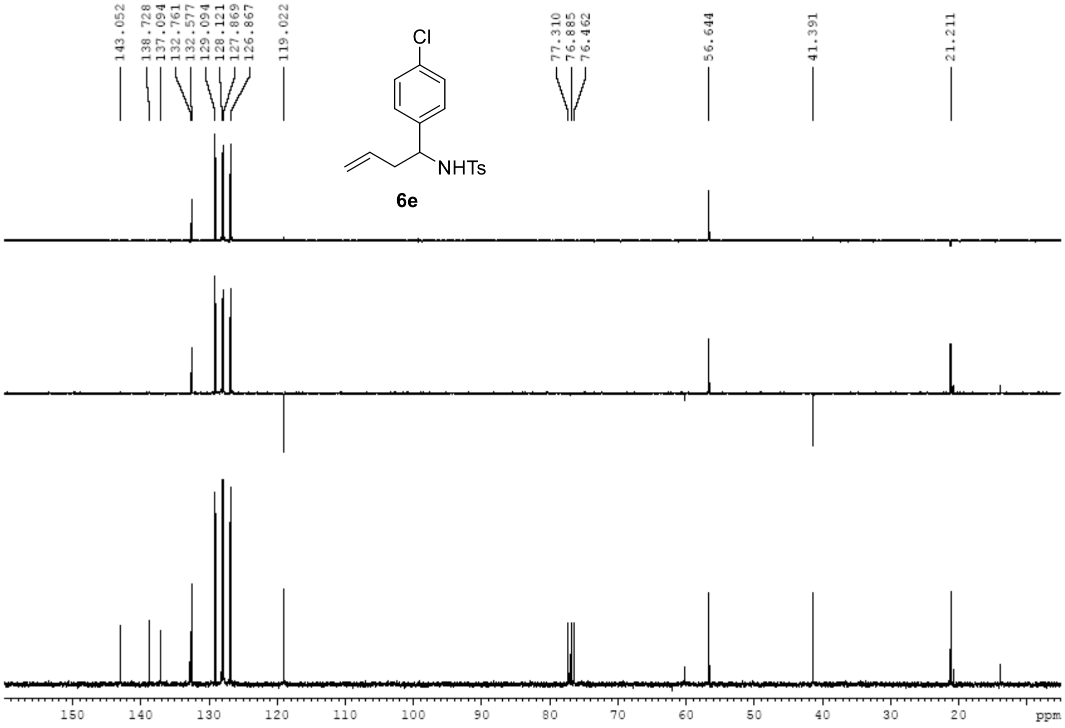

Figure S10. 13C{1H} ($CDCl_3$, 75 MHz) spectrum of compound **6e**

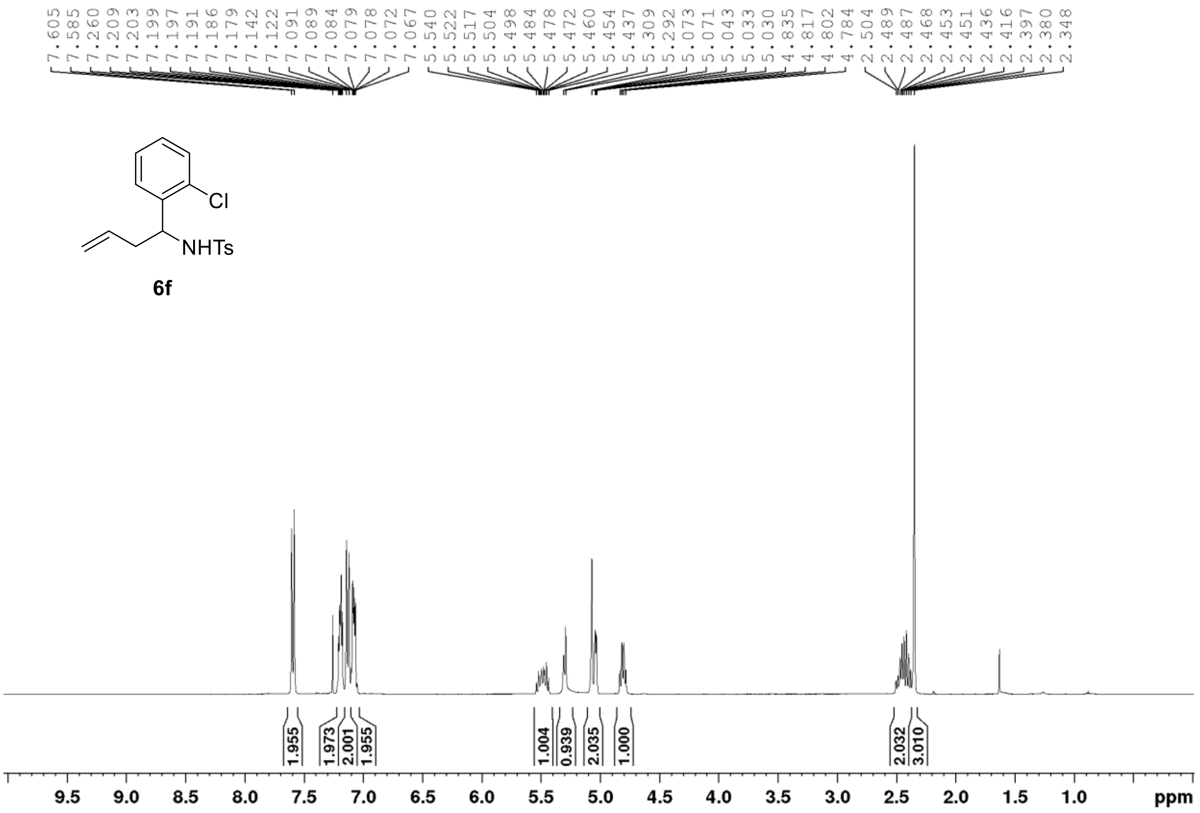

Figure S11. ^{1}H NMR ($CDCl_3$, 400 MHz) spectrum of compound **6f**

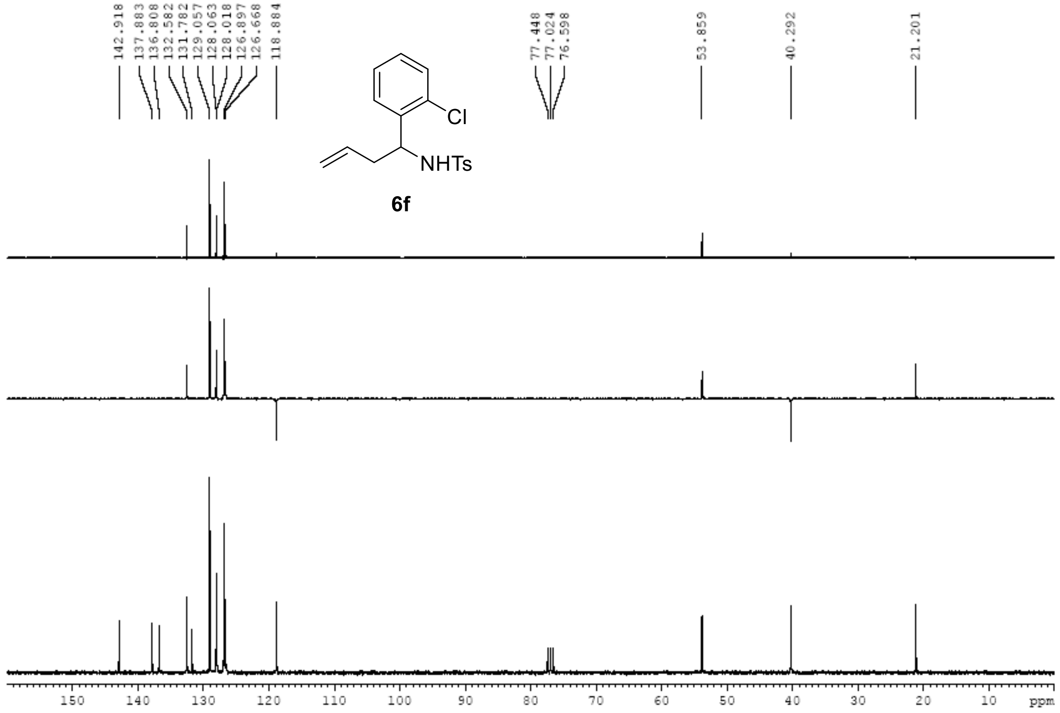

Figure S12. 13C{1H} ($CDCl_3$, 75 MHz) spectrum of compound **6f**

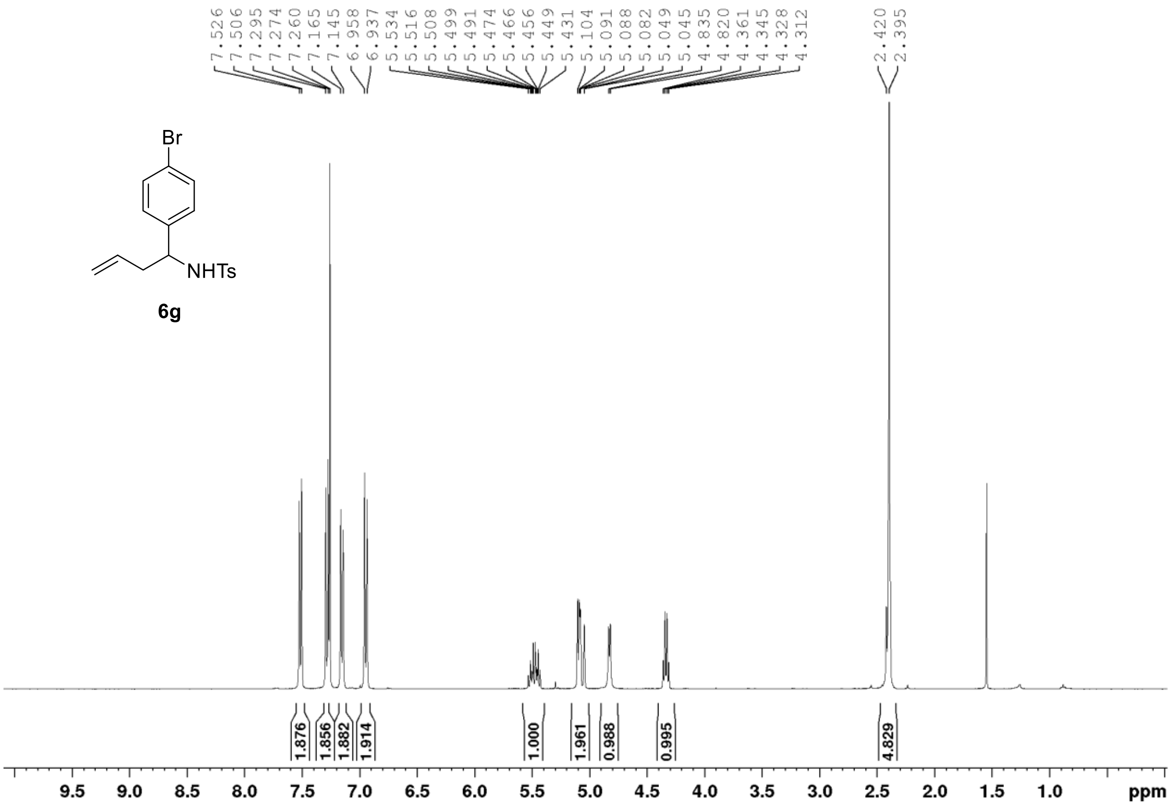

Figure S13. ^{1}H NMR ($CDCl_3$, 400 MHz) spectrum of compound **6g**

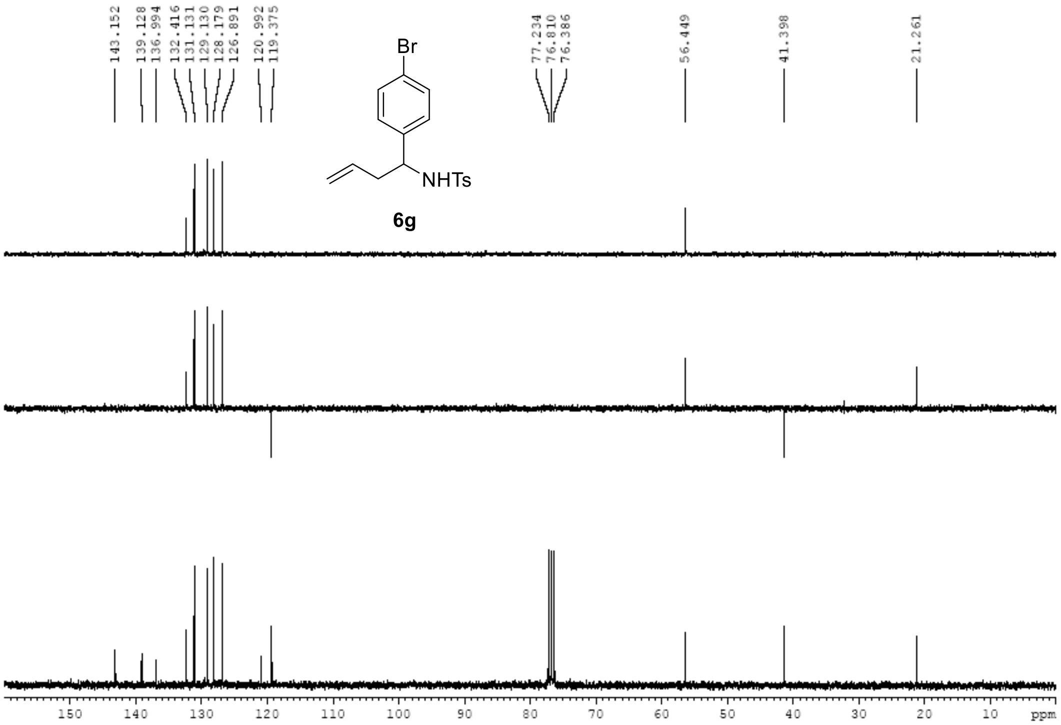

Figure S14. 13C{1H} ($CDCl_3$, 75 MHz) spectrum of compound **6g**

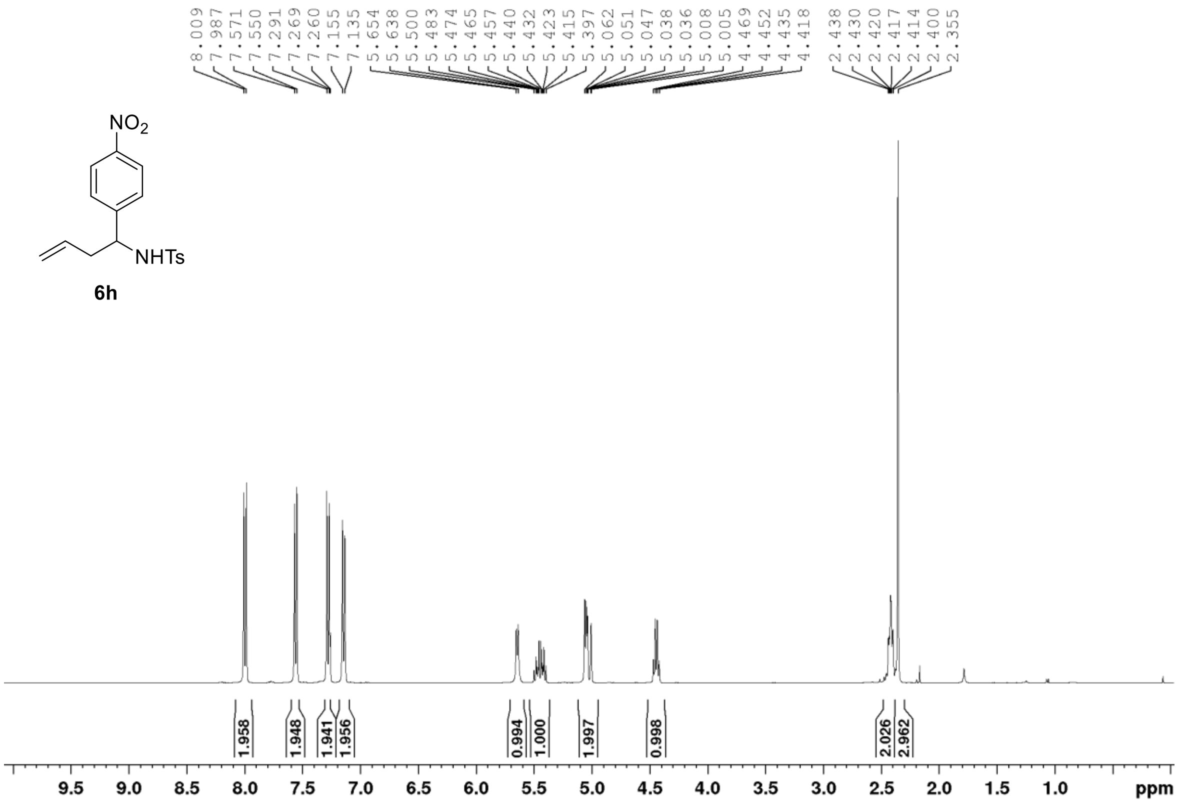

Figure S15. ^{1}H NMR ($CDCl_3$, 400 MHz) spectrum of compound 6**h**

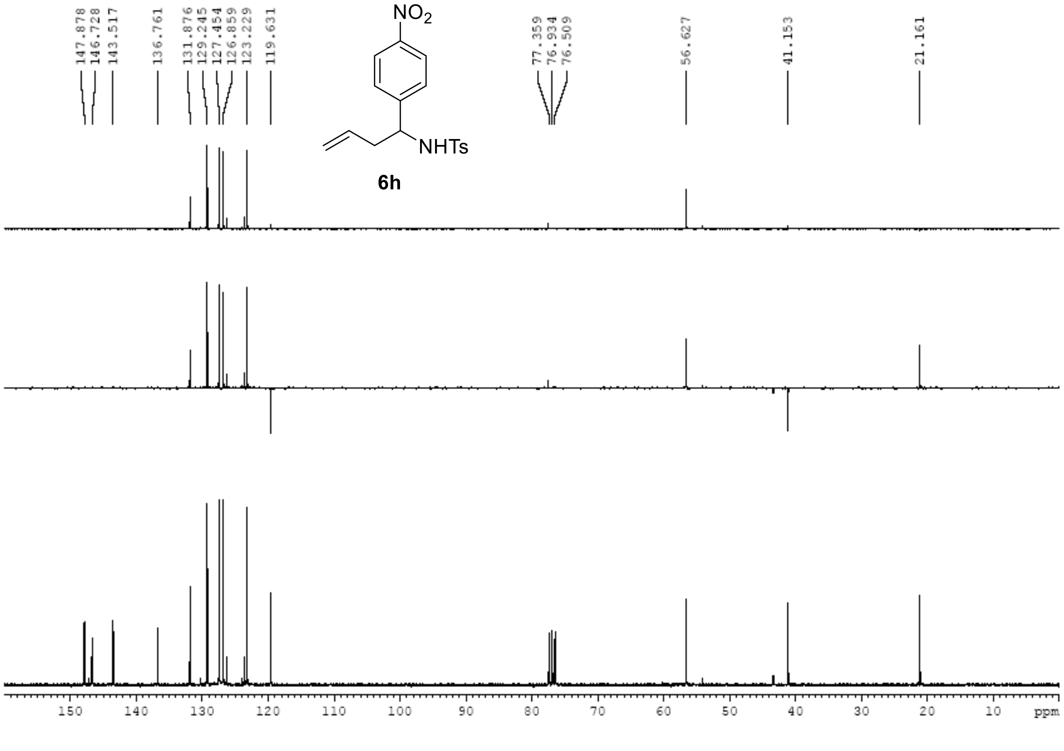

Figure S16. 13C{1H} ($CDCl_3$, 75 MHz) spectrum of compound **6h**

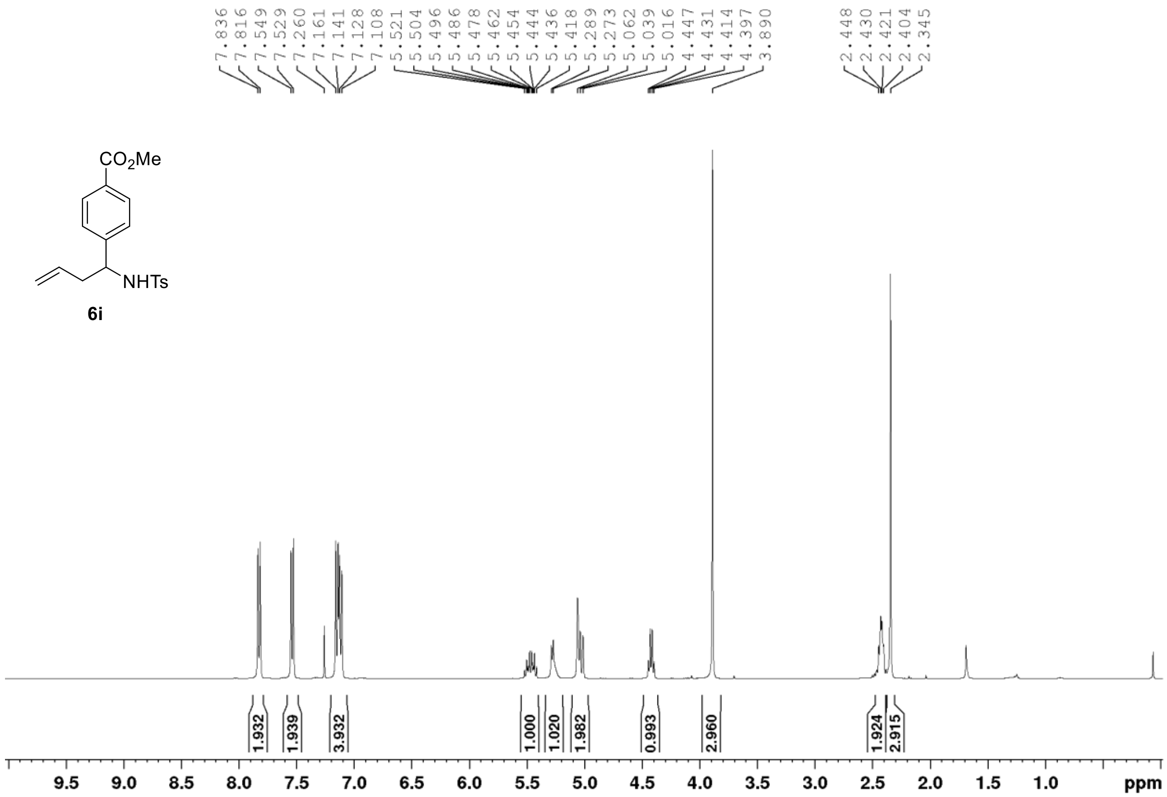

Figure S17. ^{1}H NMR ($CDCl_3$, 400 MHz) spectrum of compound **6i**

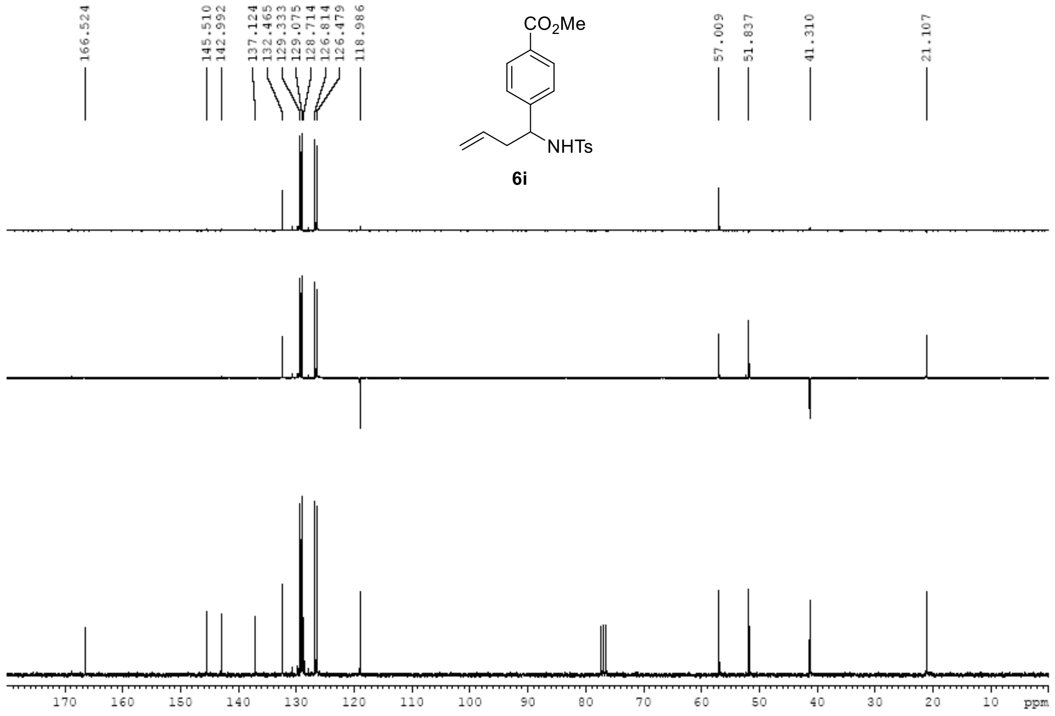

Figure S18. 13C{1H} ($CDCl_3$, 75 MHz) spectrum of compound **6i**

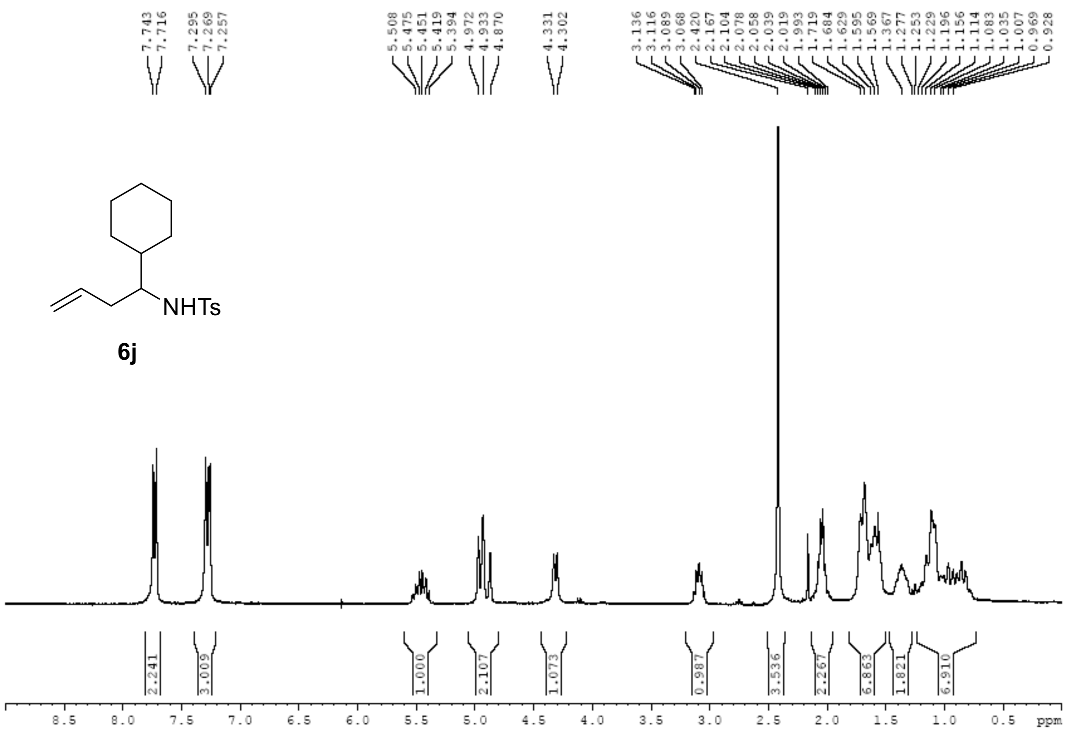

Figure S19. ^{1}H NMR ($CDCl_3$, 300 MHz) spectrum of compound **6j**

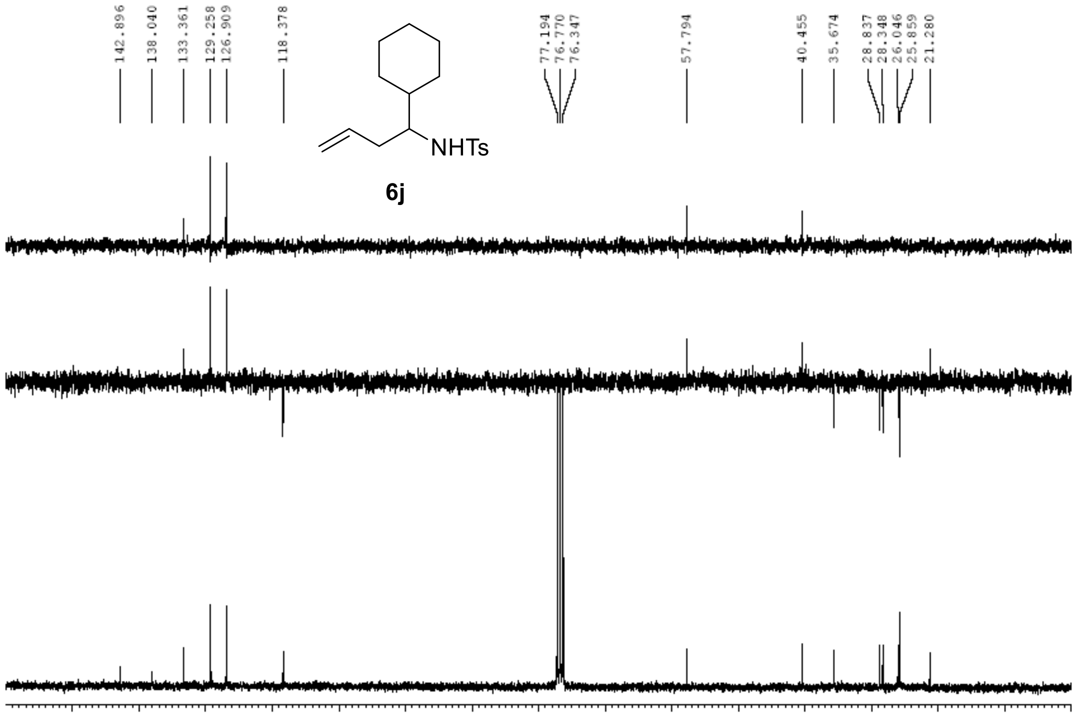

Figure S20. 13C{1H} ($CDCl_3$, 75 MHz) spectrum of compound **6j**

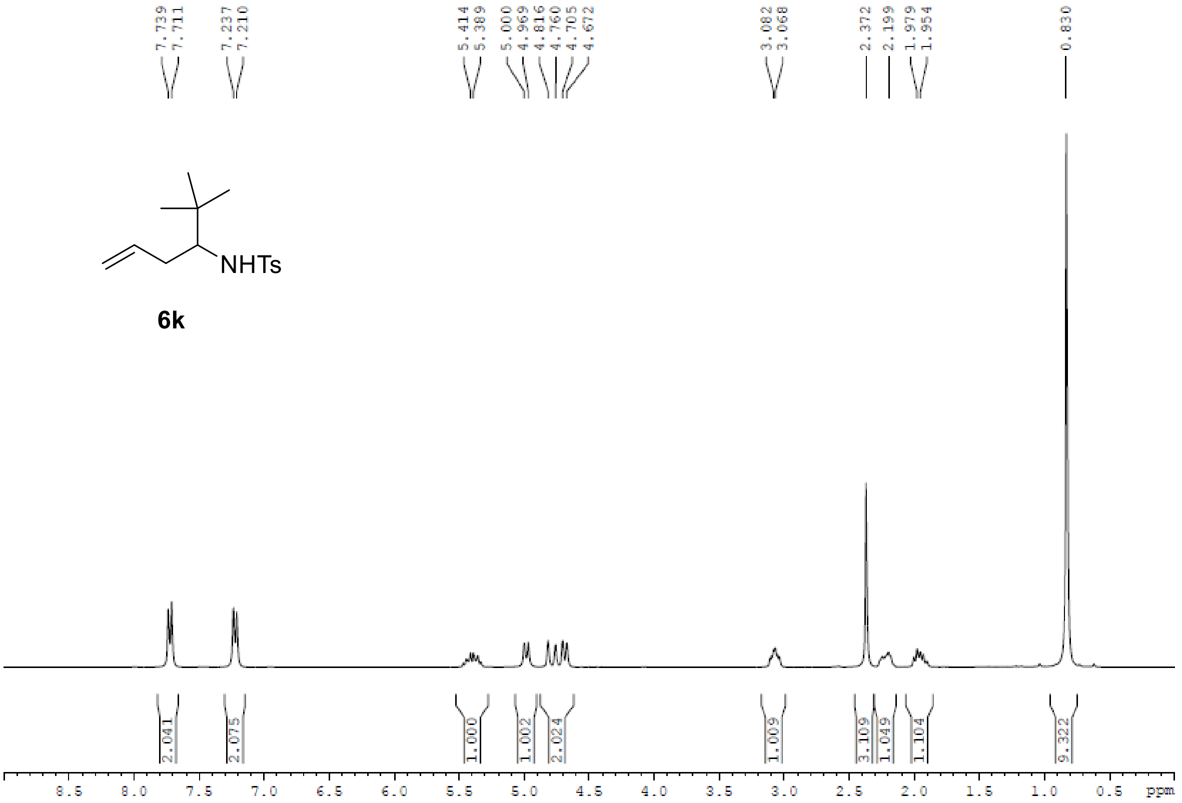

Figure S21. ^{1}H NMR ($CDCl_3$, 300 MHz) spectrum of compound **6k**

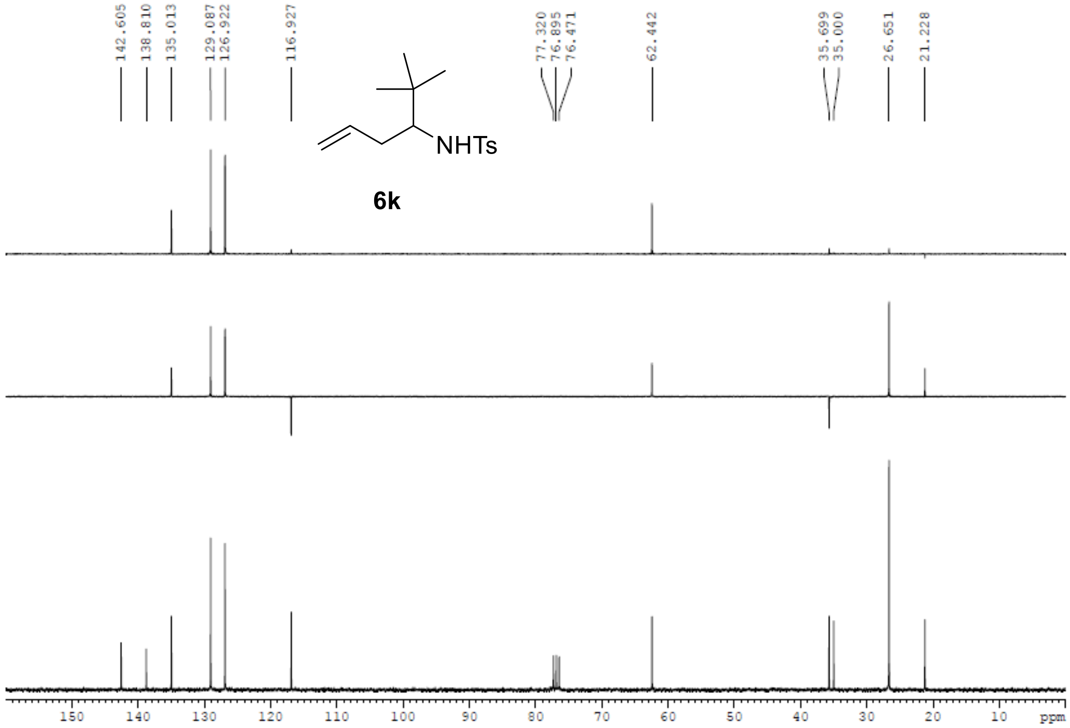

Figure S22. 13C{1H} ($CDCl_3$, 75 MHz) spectrum of compound **6k**

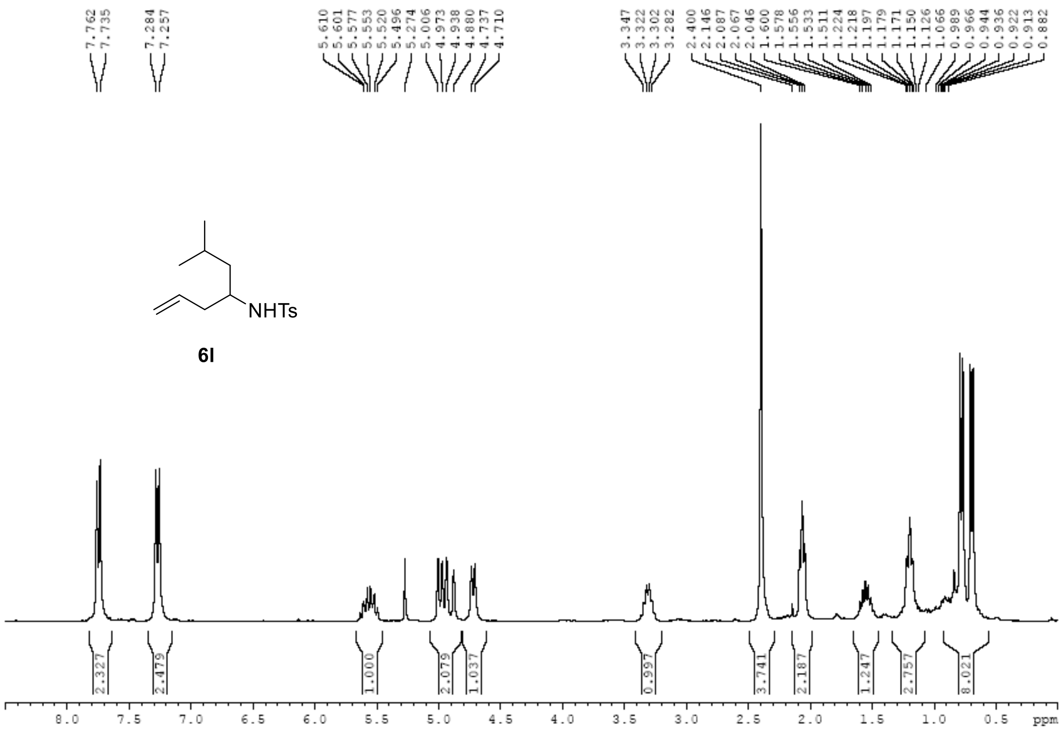

Figure S23. ^{1}H NMR ($CDCl_3$, 300 MHz) spectrum of compound **6l**

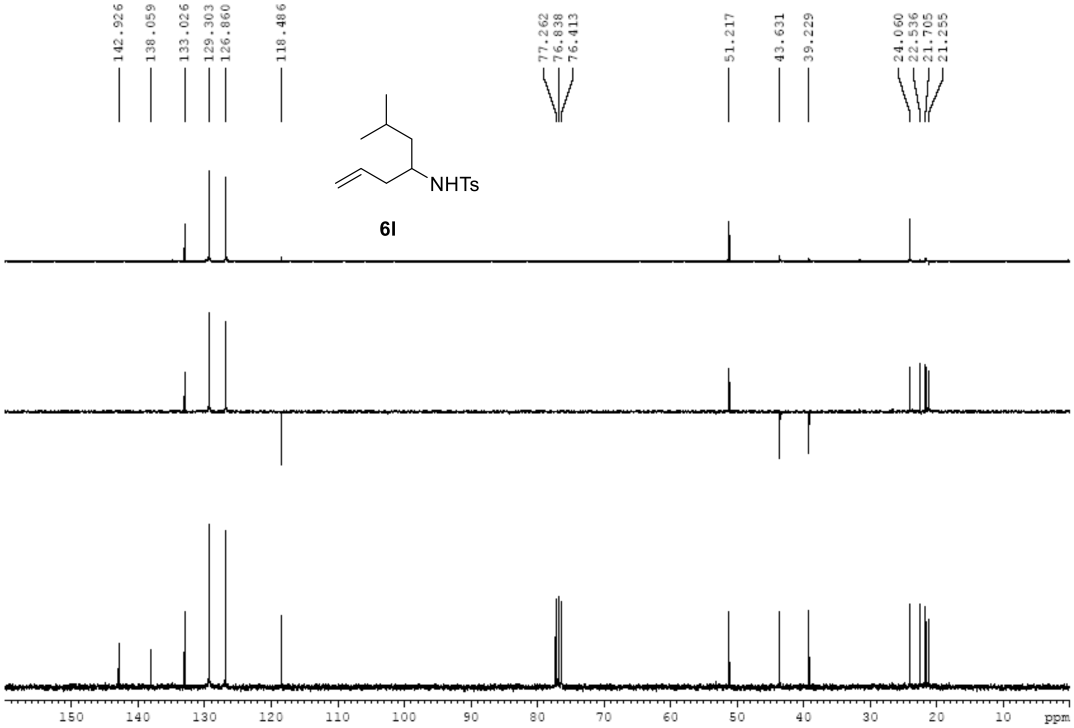

Figure S24. 13C{1H} ($CDCl_3$, 75 MHz) spectrum of compound **6l**

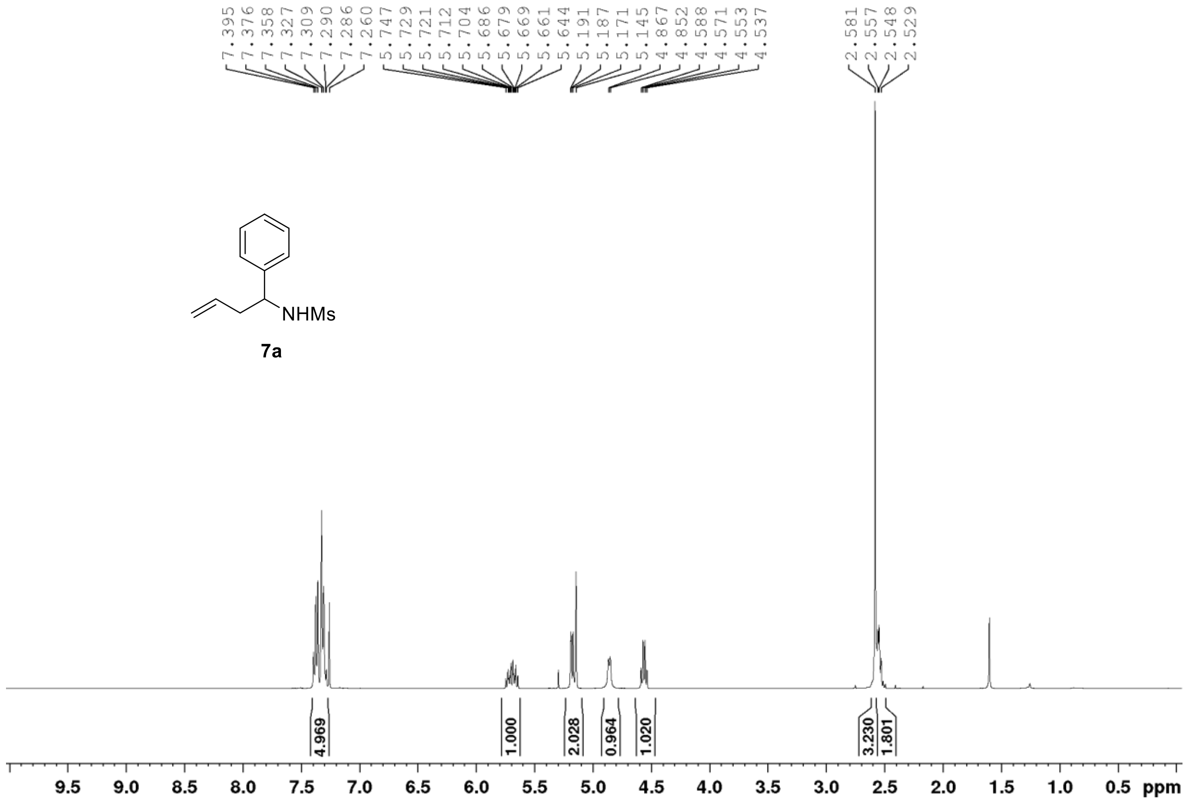

Figure S25. ^{1}H NMR ($CDCl_3$, 400 MHz) spectrum of compound **7a**

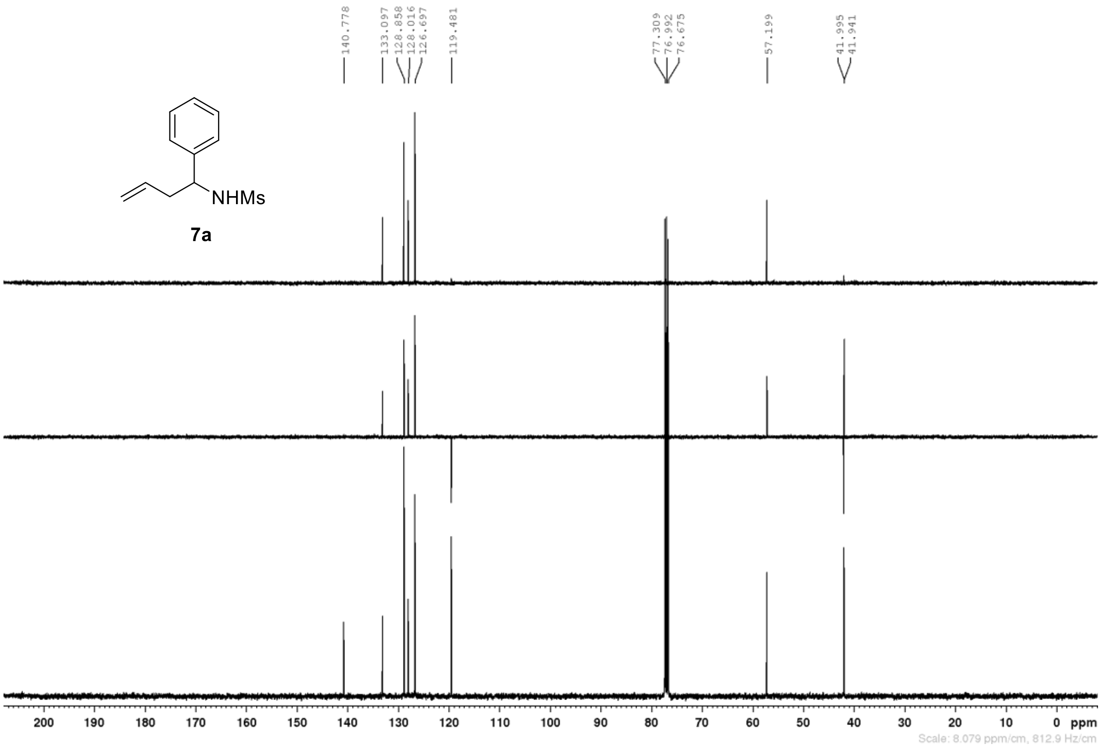

Figure S26. 13C{1H} ($CDCl_3$, 100 MHz) spectrum of compound **7a**

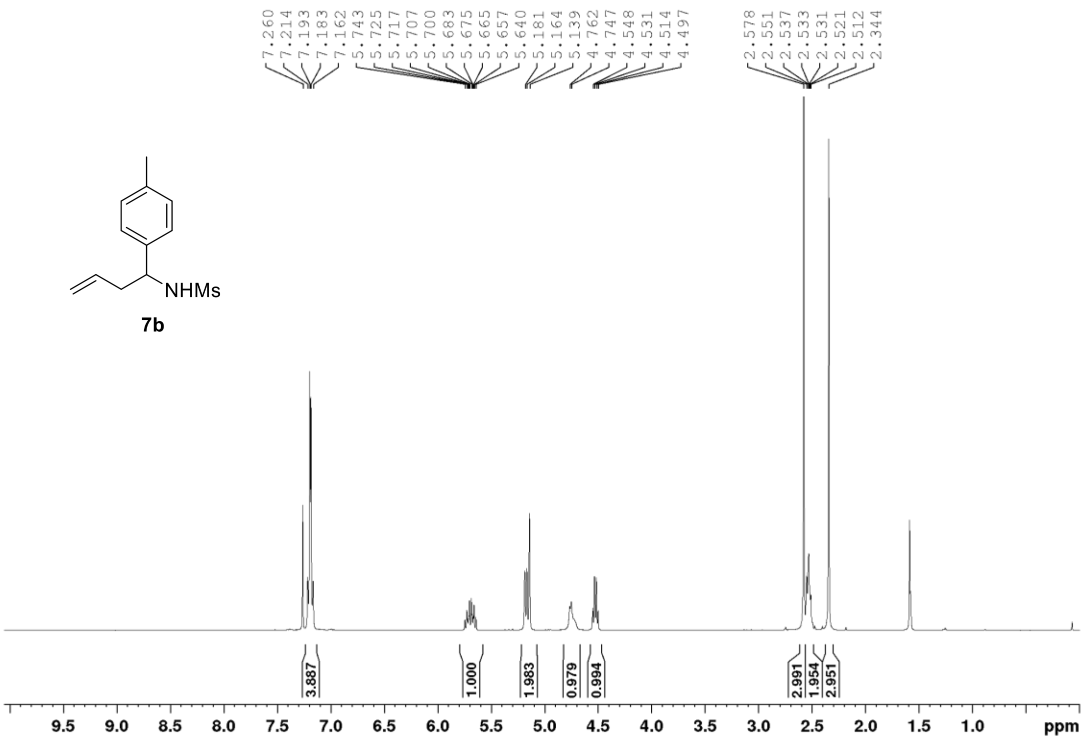

Figure S27. ¹H NMR ($CDCl_3$, 400 MHz) spectrum of compound **7b**

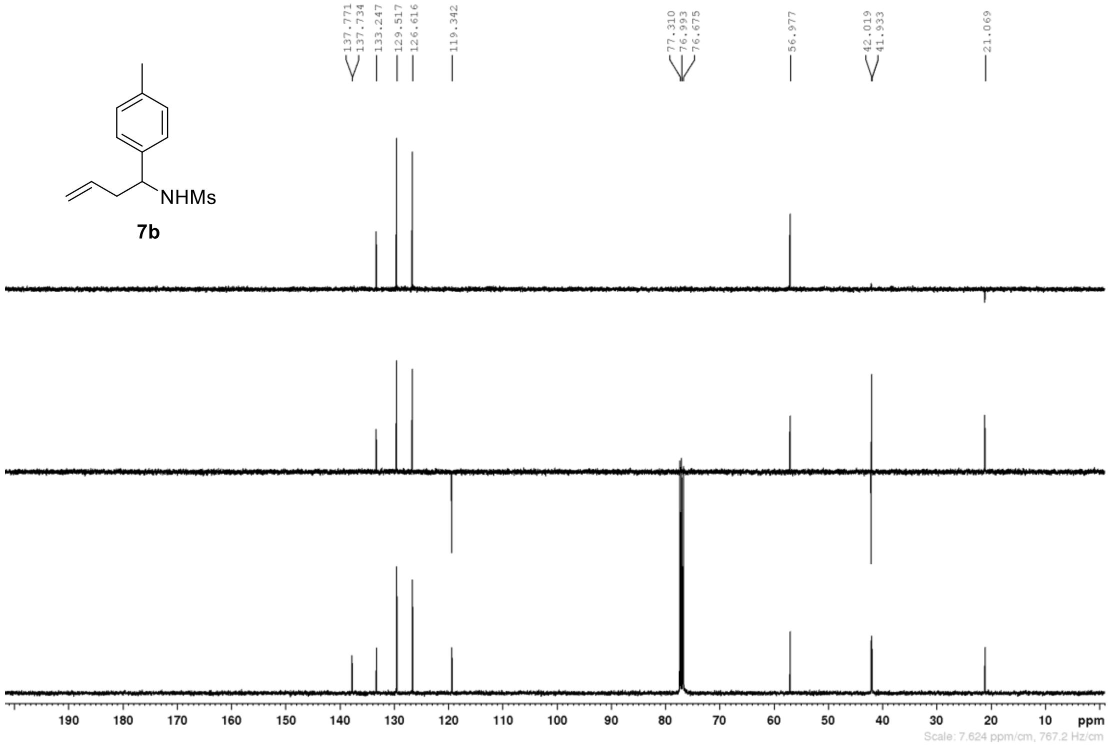

Figure S28. 13C{1H} ($CDCl_3$, 100 MHz) spectrum of compound **7b**

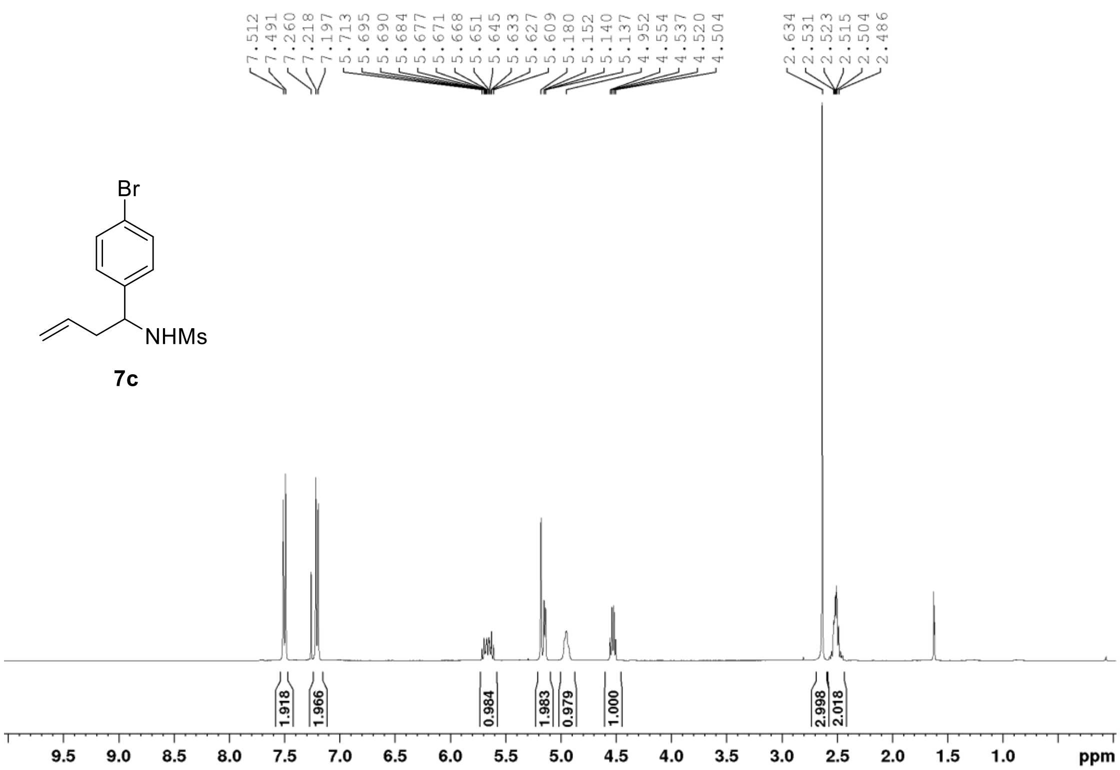

Figure S29. ^{1}H NMR (CDCl$_3$, 400 MHz) spectrum of compound **7c**

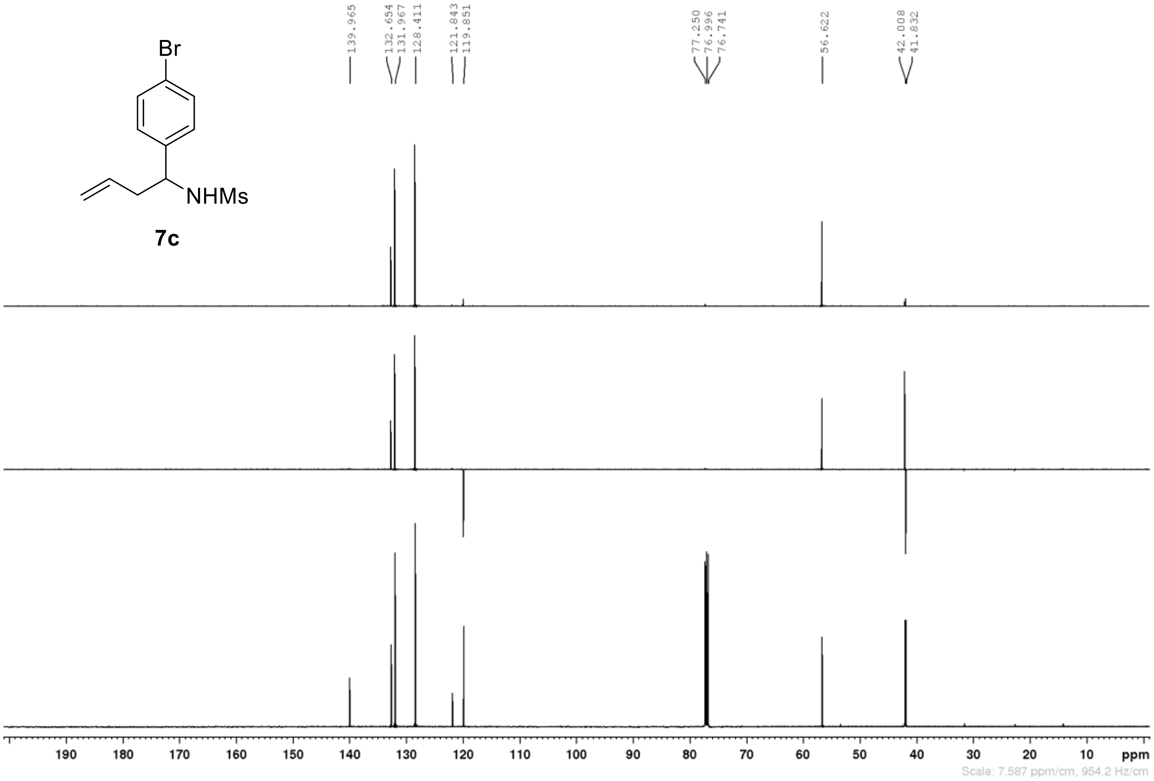

Figure S30. 13C{1H} (CDCl$_3$, 125 MHz) spectrum of compound **7c**

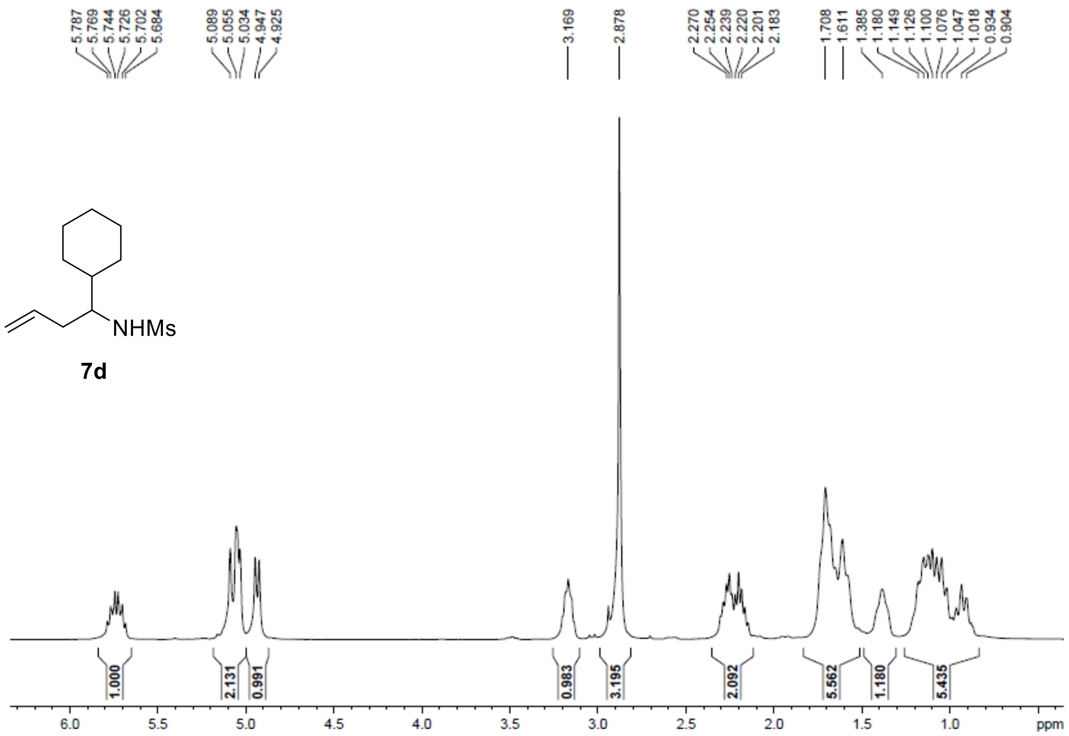

Figure S31. ^{1}H NMR ($CDCl_3$, 400 MHz) spectrum of compound **7d**

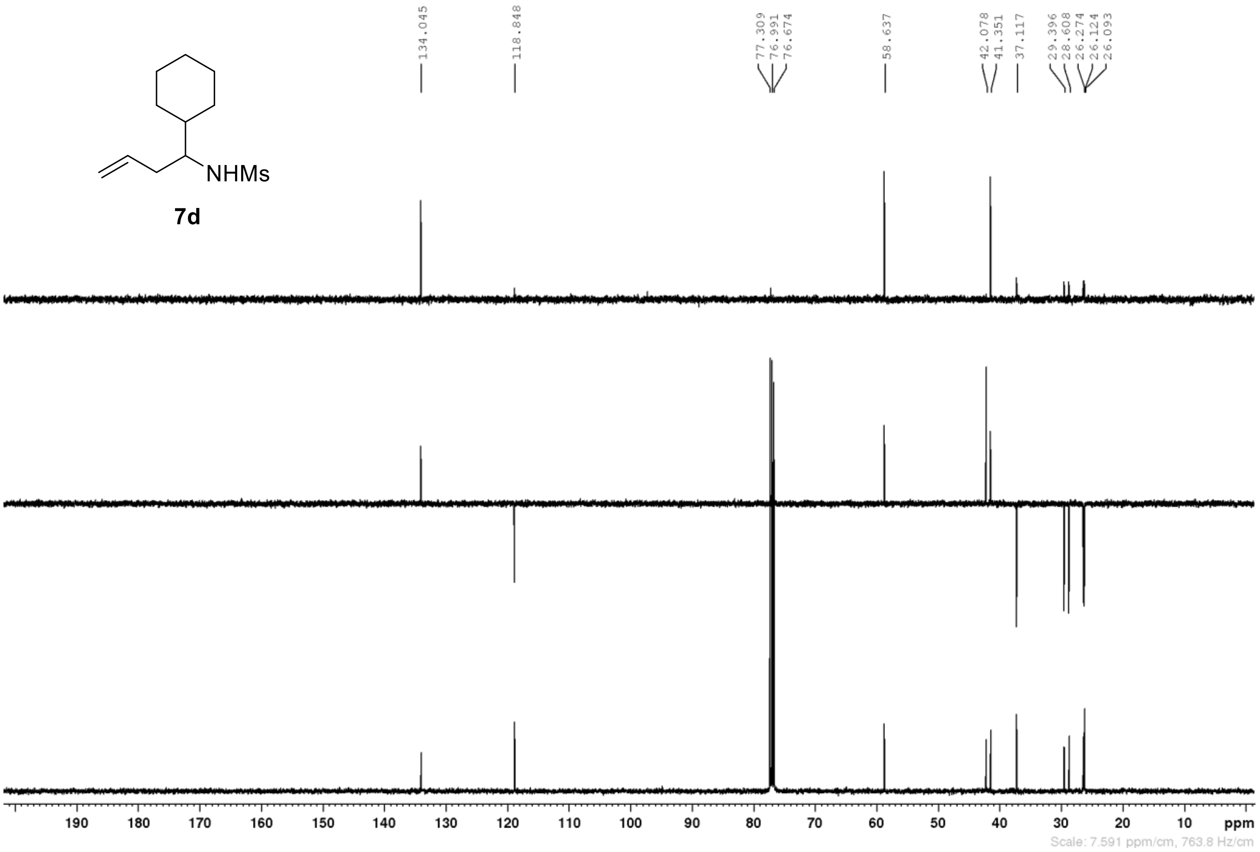

Figure S32. 13C{1H} ($CDCl_3$, 100 MHz) spectrum of compound **7d**

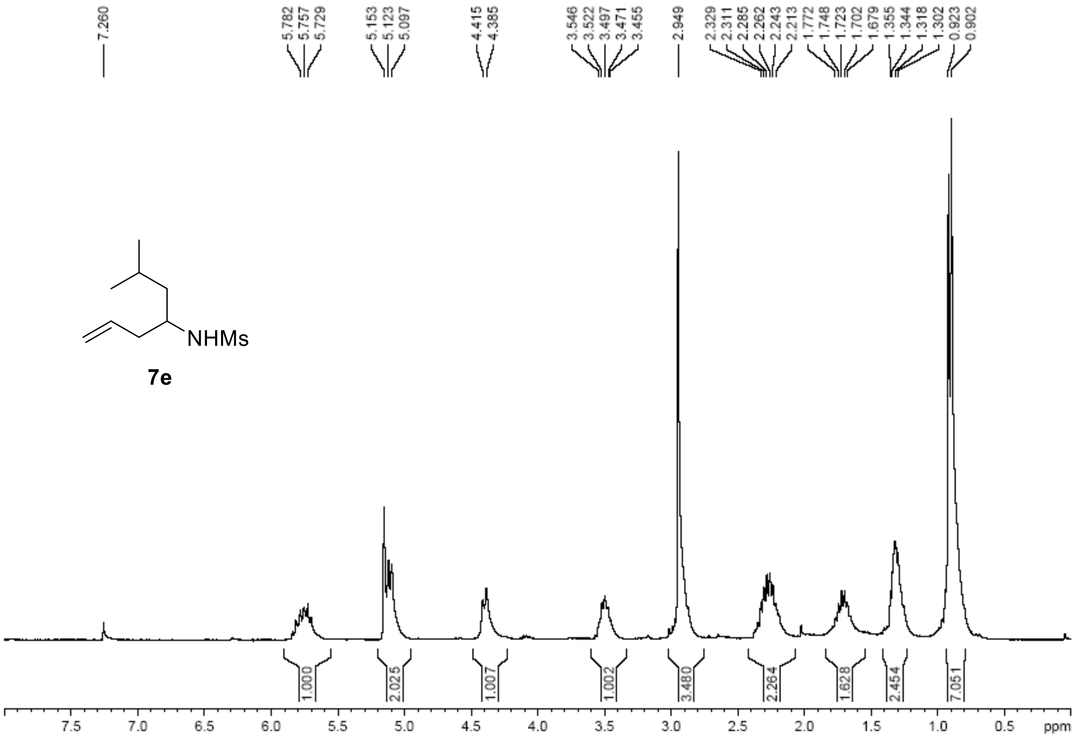

Figure S33. ^{1}H NMR ($CDCl_3$, 300 MHz) spectrum of compound **7e**

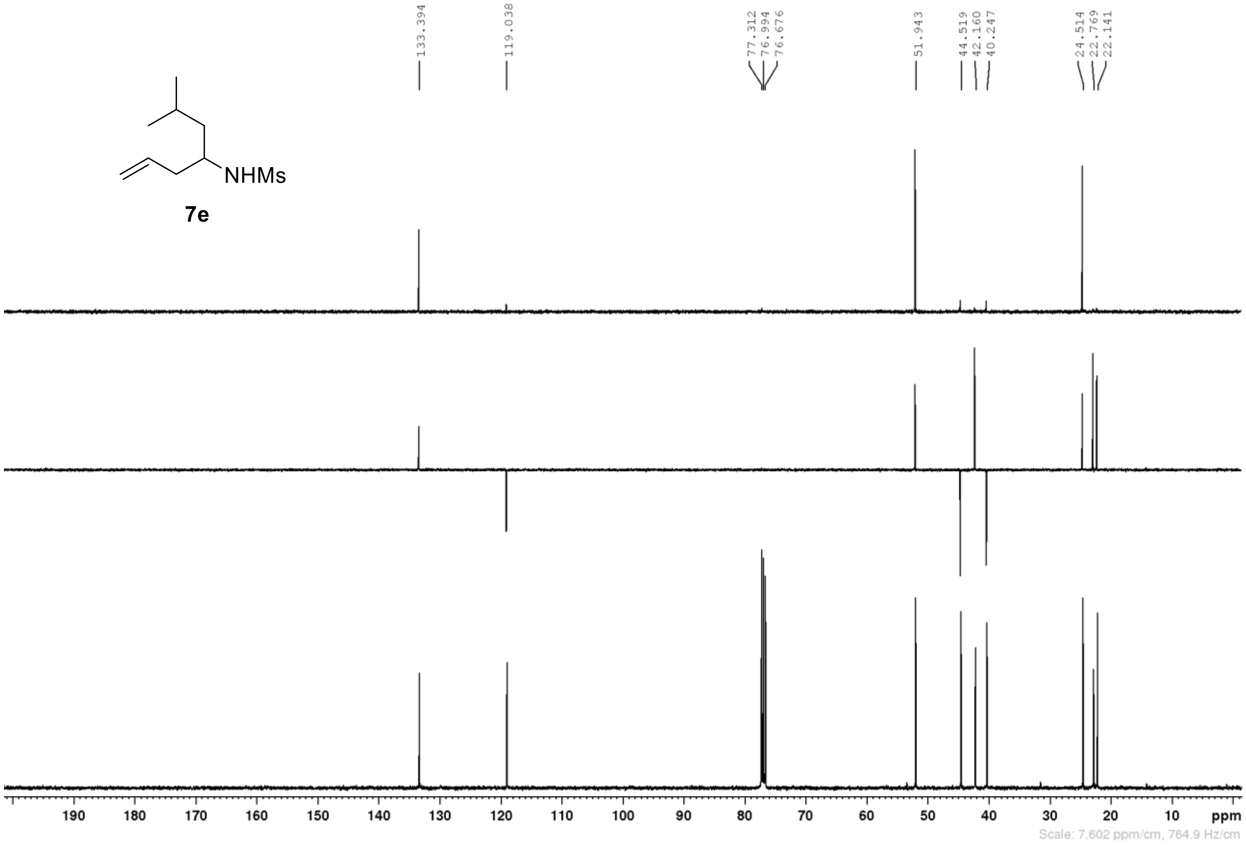

Figure S34. 13C{1H} ($CDCl_3$, 100 MHz) spectrum of compound **7e**

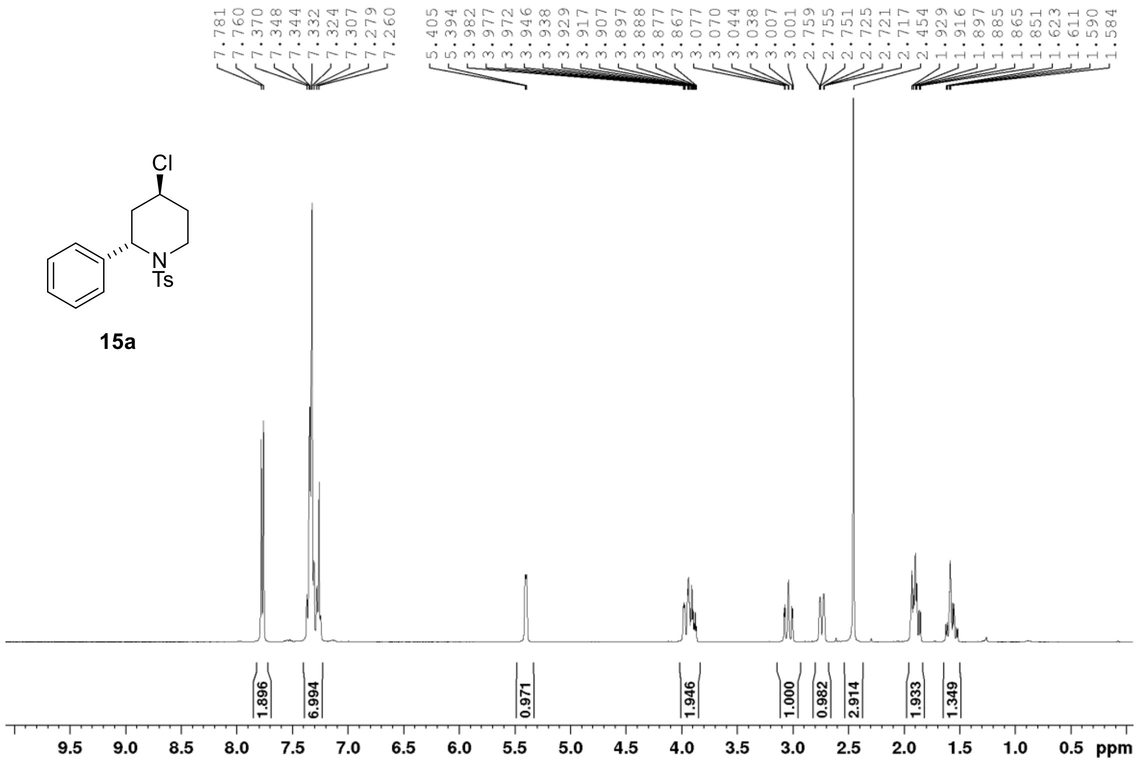

Figure S35. ^{13}H NMR ($CDCl_3$, 400 MHz) spectrum of compound **15a**

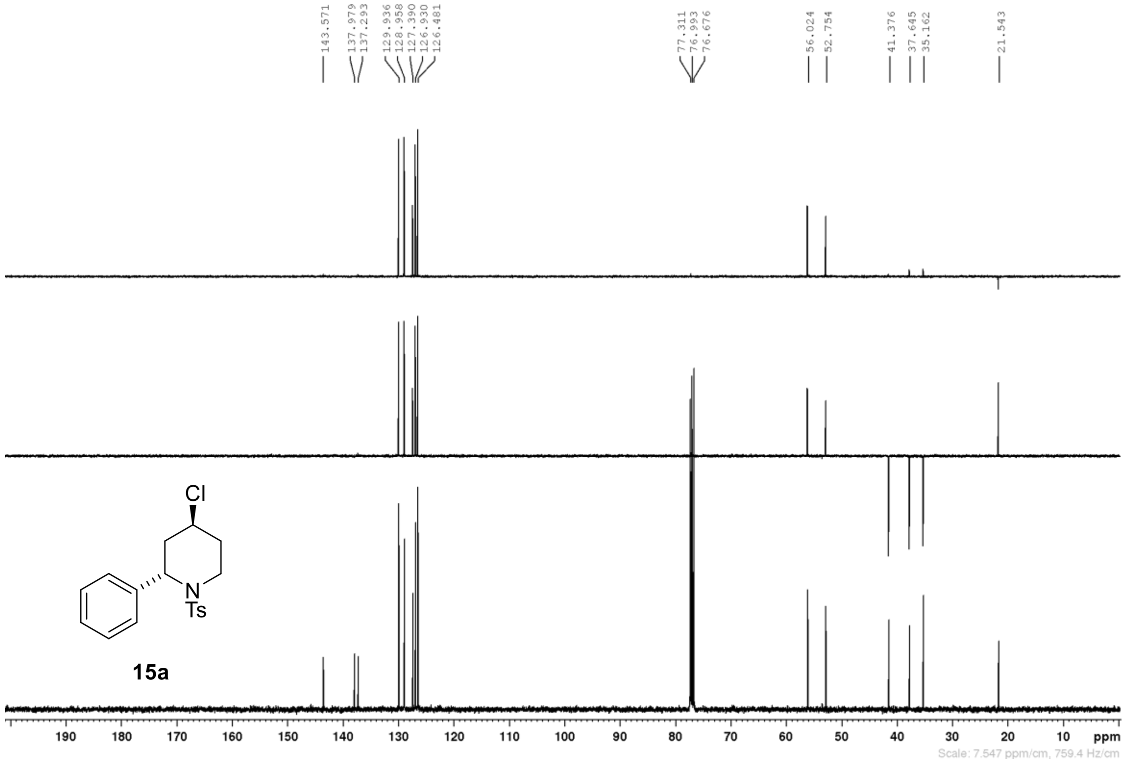

Figure S36. 13C{1H} ($CDCl_3$, 100 MHz) spectrum of compound **15a**

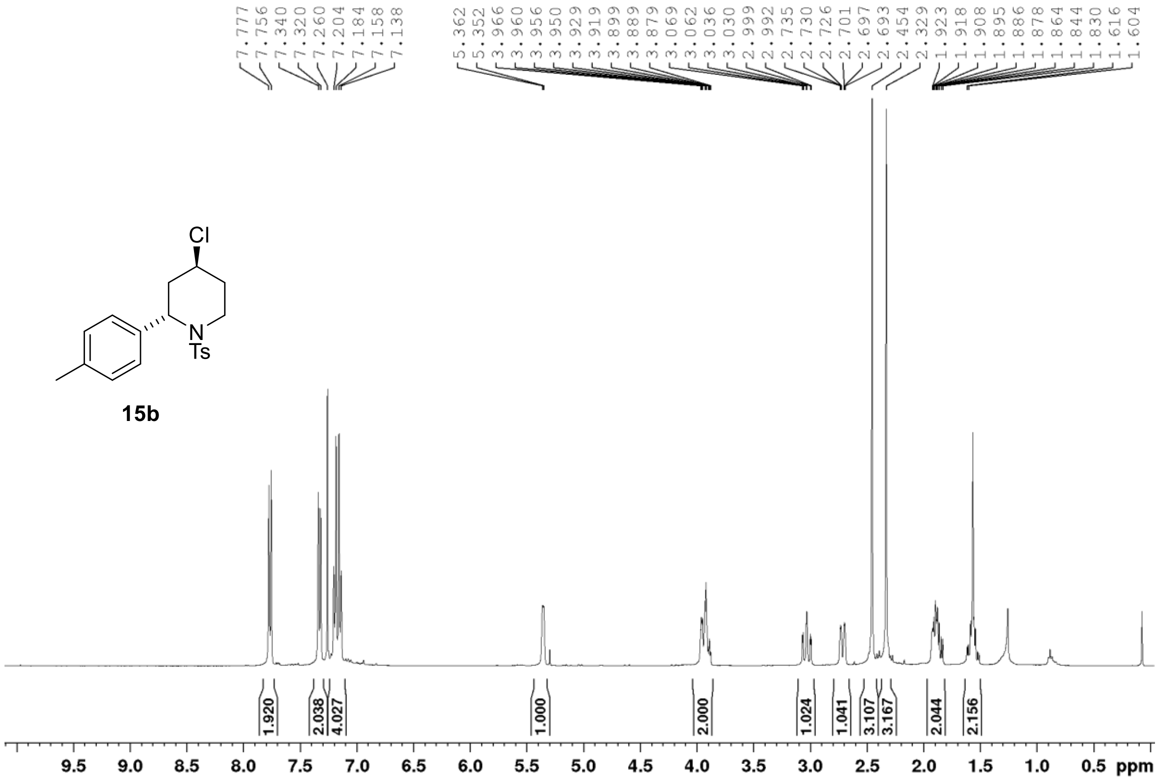

Figure S37. 13H NMR ($CDCl_3$, 400 MHz) spectrum of compound **15b**

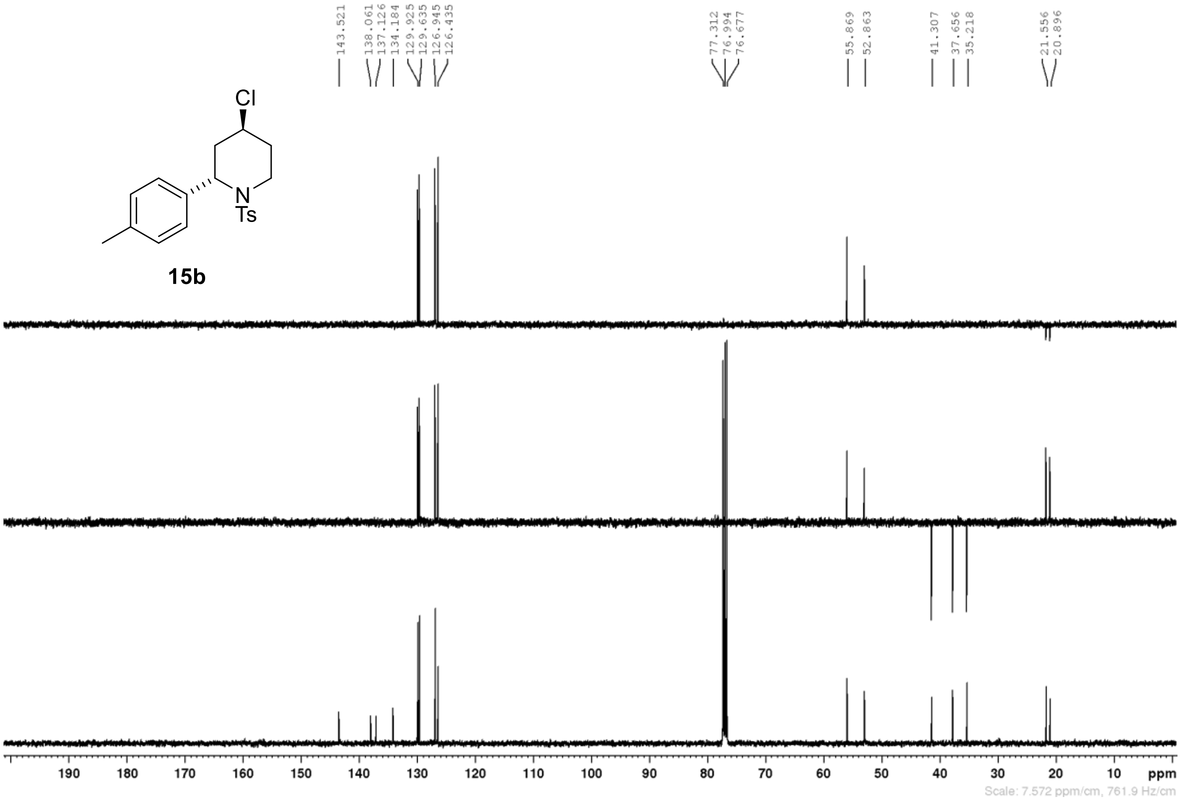

Figure S38. 13C{1H} ($CDCl_3$, 100 MHz) spectrum of compound **15b**

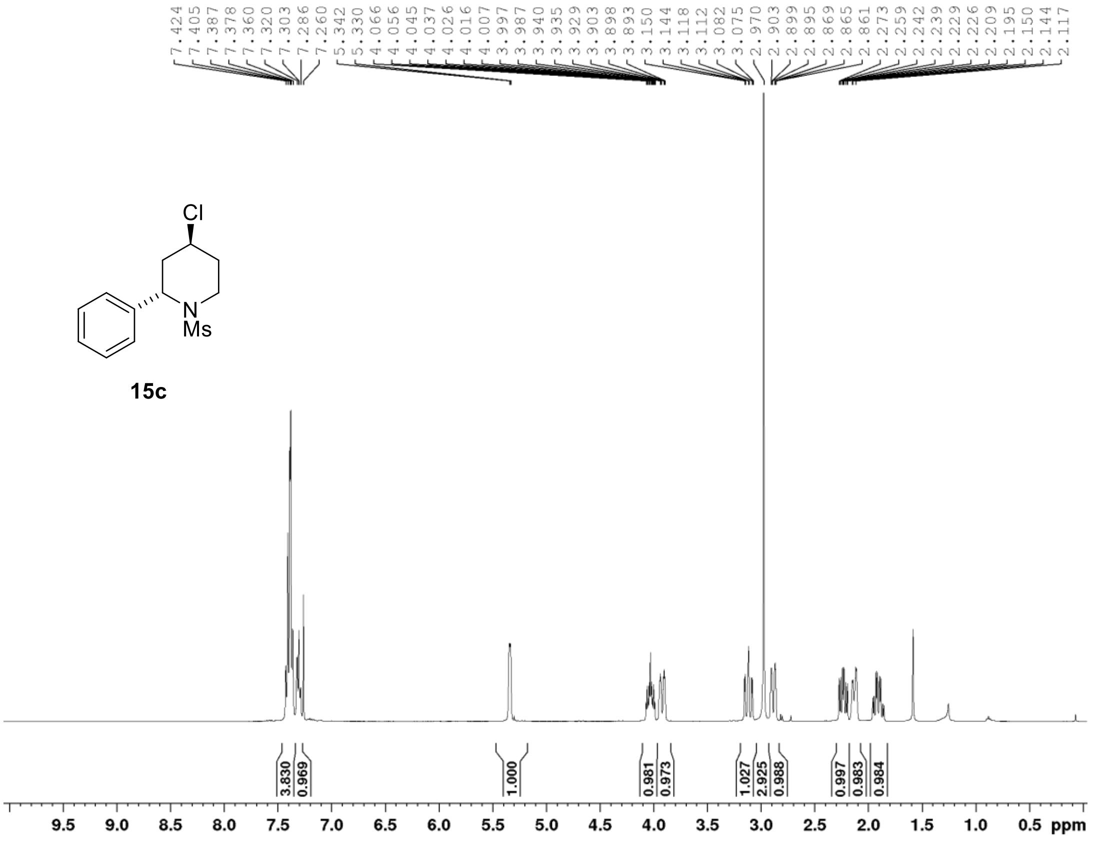

Figure S39. [13]H NMR ($CDCl_3$, 400 MHz) spectrum of compound **15c**

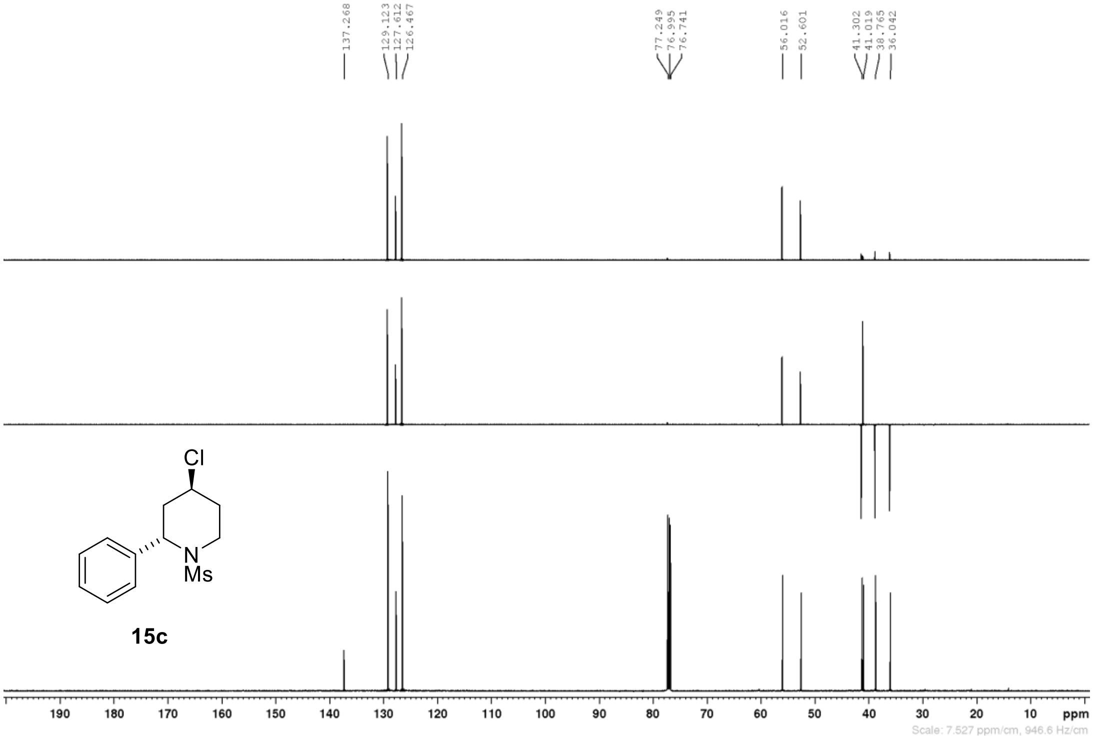

Figure S40. 13C{1H} ($CDCl_3$, 100 MHz) spectrum of compound **15c**

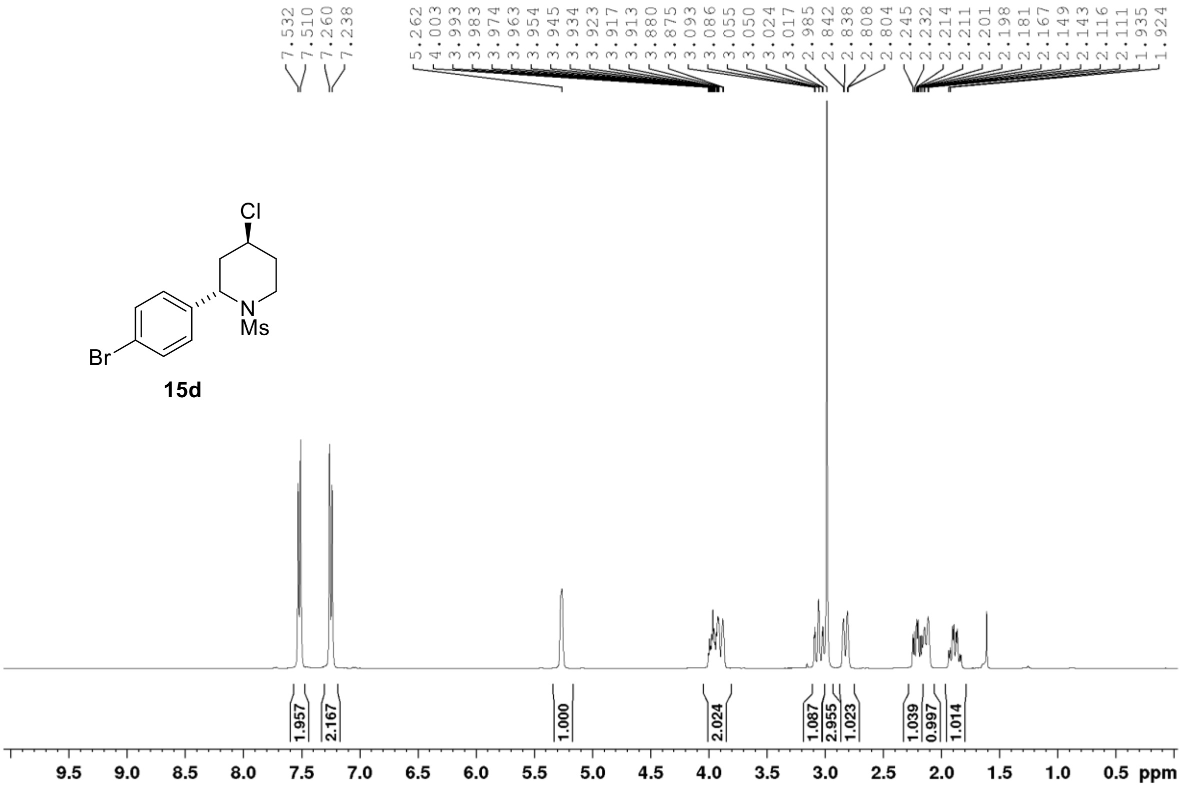

Figure S41. [13]H NMR ($CDCl_3$, 400 MHz) spectrum of compound **15d**

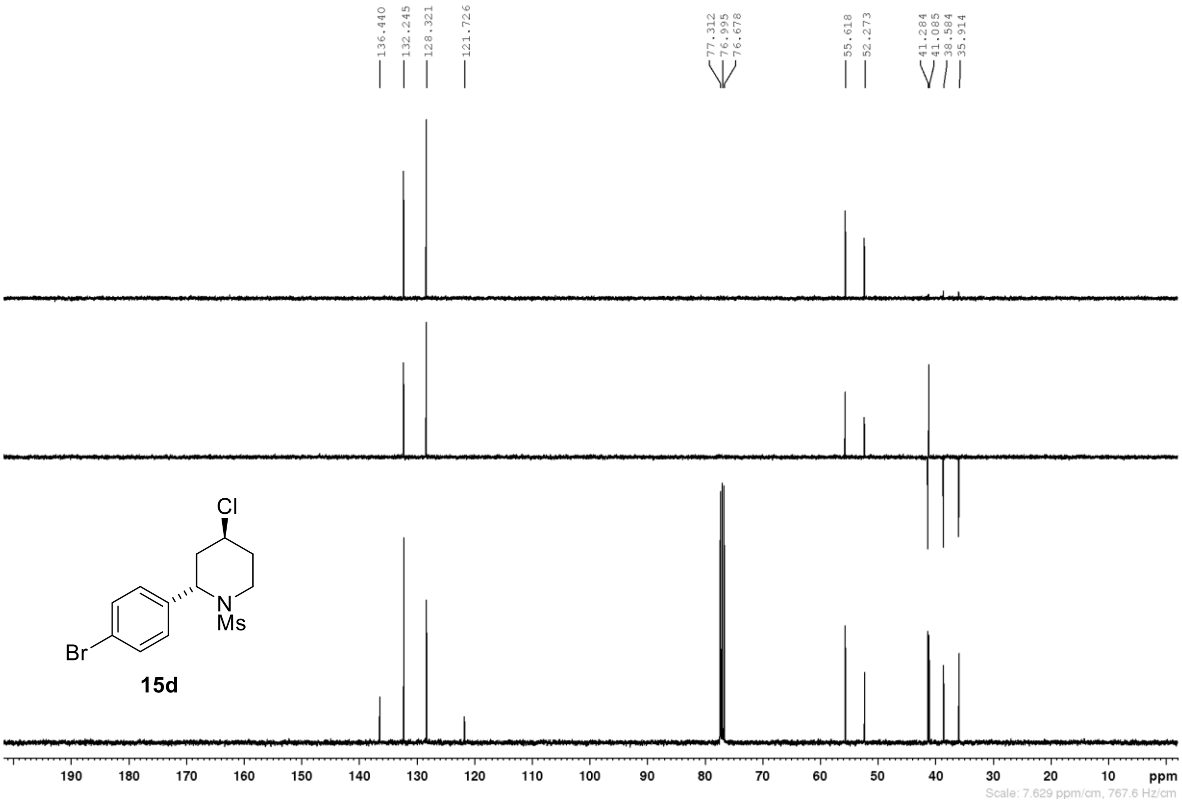

Figure S42. 13C{1H} ($CDCl_3$, 100 MHz) spectrum of compound **15d**

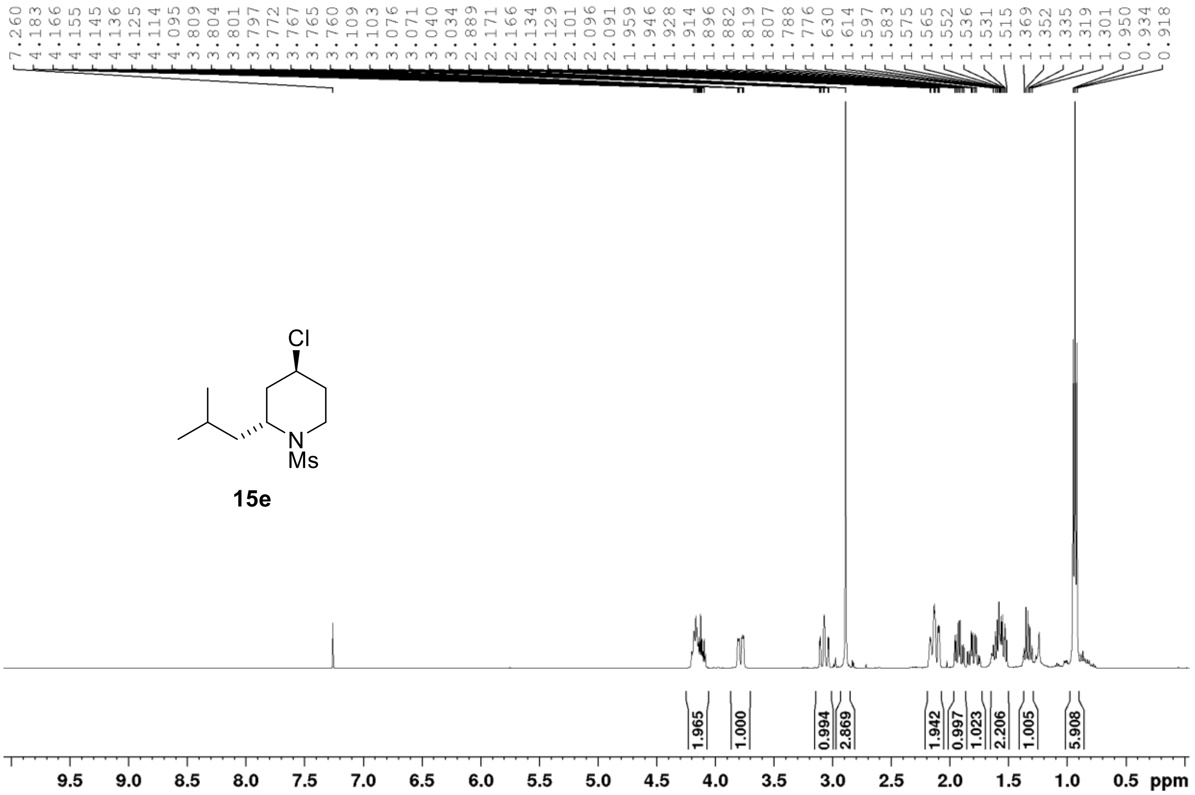

Figure S43. [13]H NMR ($CDCl_3$, 400 MHz) spectrum of compound **15e**

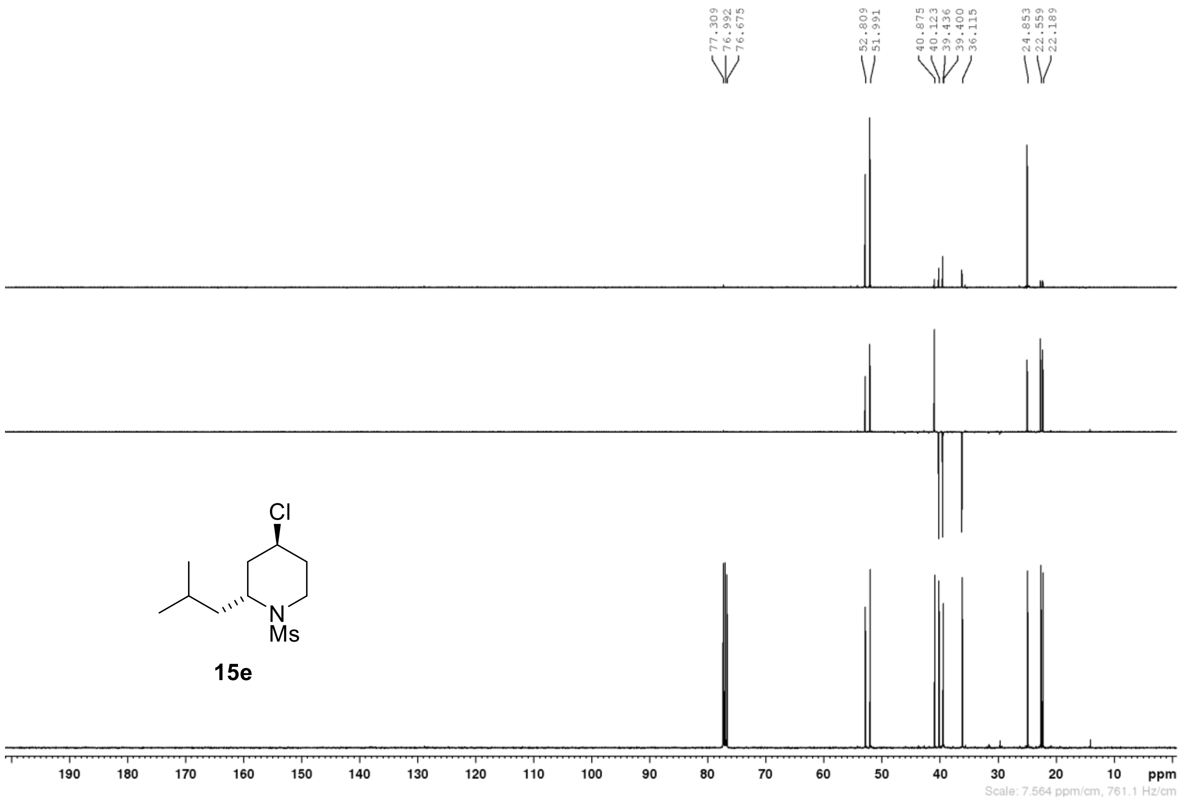

Figure S44. 13C{1H} ($CDCl_3$, 100 MHz) spectrum of compound **15e**

Lipophilicity and *in vitro* antiproliferative data

Here we present the full study about the lipophilicity and *in vitro* antiproliferative properties of the homoallyl sulfonylamides.

Table 3. Lipophilicity and *in vitro* antiproliferative activity of the homoallyl sulfonylamides against human solid tumor cells.

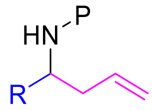

6a-6l, 7a-7e

Entry[1]	Comp	ClogP	P	R	A 2780	HBL-100	HeLa	SW1573	T-47D	WiDr
1	6a	4.07	Ts	Ph	30±17	34±3	48±6	26±8	59±35	51±8
2	6b	5.24	Ts	Napf	15±2	18±1	20±4	15±3	18±0.2	24±5
3	6c	4.57	Ts	*p*-MePh	>100	>100	>100	>100	>100	>100
4	6d	4.21	Ts	*p*-FPh	19±9	27±1	29±7	19±4	23±2	29±8
5	6e	4.78	Ts	*p*-ClPh	18±6	23±2	25±8	27±8	24±3	33±9
6	6f	4.78	Ts	*o*-ClPh	>100	>100	>100	>100	>100	>100
7	6g	4.93	Ts	*p*-BrPh	>100	>100	>100	>100	>100	>100
8	6h	3.81	Ts	*p*-NO_2Ph	12±3	31±9	46±22	19±4	24±2	32±0.6
9	6i	4.04	Ts	*p*-MeO_2CPh	>100	>100	>100	>100	>100	>100
10	6j	4.95	Ts	*c*-Hex	14±2	16±4	20±3	14±2	20±4	22±3
11	6k	4.12	Ts	*t*-Bu	>100	>100	>100	>100	>100	>100
12	6l	4.29	Ts	*i*-Bu	21±9	21±4	26±1	14±4	25±4	31±3
13	7a	1.69	Ms	Ph	>100	>100	>100	>100	>100	>100
14	7b	2.19	Ms	*p*-MePh	>100	>100	>100	>100	>100	>100
15	7c	2.56	Ms	*p*-BrPh	>100	>100	>100	>100	>100	>100
16	7d	2.66	Ms	*c*-Hex	>100	>100	>100	>100	>100	>100
17	7e	2.00	Ms	*i*-Bu	>100	>100	>100	>100	>100	>100

[1] Values of GI_{50} ± standard deviation, in μM. Means of two to four independent experiments.

During the biological testing stage, lipophilicity and *in vitro* antiproliferative activity were measured. The amine-protecting group provided the first quote regarding the SAR, since all the active compounds bear the tosyl group (Table 3, entries 1, 2, 4, 5, 8, 10 and 12), **6b** and **6j** showing the best antiproliferative performance. The next group includes **6d**, **6e** and **6l**, the activity of the latter derivative being rather similar to that of the two halogenated derivatives but having some special selectivity for the line SW1573. Compound **6a** showed the worst activity profile but also its performance was highly dependent on the cell line. Finally, the structure **6h** provided a mixed outcome where its activity value against the cell line A2780 was the best in the series, whilst HBL-100 and HeLa appeared to be more resistant to its action. Additionally, **6h** showed similar antiproliferative active **6d** for cell lines SW1573, T-47D and WiDr. Measurements of lipophilicity ranged from 3.81 to 5.24 for active compounds, which is not a significant difference. No correlation was found between bioactivity profiles and lipophilicity values.

Computational details

Geometry optimizations of the molecules were performed without symmetry constraints using the Gaussian-09 (RevD.01)[1] suite of programs and the hybrid meta-GGA M06-2X functional[2] in conjunction with the triple-ζ basis-set def2-SVP[3] basis set. This level of theory has been proven to provide accurate results for organic chemistry reactions.[4] Solvent effects (solvent = dichloromethane) were taken into account with the Polarization Continuum Model (PCM) method.[5] This level is denoted PCM(CH_2Cl_2)-M06-2X/def2-SVP. Reactants and adducts were characterized by frequency calculations, and have positive definite Hessian matrices. Transition states (TS's) show only one negative eigenvalue in their diagonalized force constant matrices, and their associated eigenvectors were confirmed to correspond to the motion along the reaction coordinate under consideration using the Intrinsic Reaction Coordinate (IRC) method.[6]

[1] Gaussian 09, Revision D.01, Frisch, M. J.; Trucks, G. W.; Schlegel, H. B.; Scuseria, G. E.; Robb, M. A.; Cheeseman, J. R.; Scalmani, G.; Barone, V.; Mennucci, B.; Petersson, G. A.; Nakatsuji, H.; Caricato, M.; Li, X.; Hratchian, H. P.; Izmaylov, A. F.; Bloino, J.; Zheng, G.; Sonnenberg, J. L.; Hada, M.; Ehara, M.; Toyota, K.; Fukuda, R.; Hasegawa, J.; Ishida, M.; Nakajima, T.; Honda, Y.; Kitao, O.; Nakai, H.; Vreven, T.; Montgomery, J. A., Jr.; Peralta, J. E.; Ogliaro, F.; Bearpark, M.; Heyd, J. J.; Brothers, E.; Kudin, K. N.; Staroverov, V. N.; Kobayashi, R.; Normand, J.; Raghavachari, K.; Rendell, A.; Burant, J. C.; Iyengar, S. S.; Tomasi, J.; Cossi, M.; Rega, N.; Millam, J. M.; Klene, M.; Knox, J. E.; Cross, J. B.; Bakken, V.; Adamo, C.; Jaramillo, J.; Gomperts, R.; Stratmann, R. E.; Yazyev, O.; Austin, A. J.; Cammi, R.; Pomelli, C.; Ochterski, J. W.; Martin, R. L.; Morokuma, K.; Zakrzewski, V. G.; Voth, G. A.; Salvador, P.; Dannenberg, J. J.; Dapprich, S.; Daniels, A. D.; Farkas, Ö.; Foresman, J. B.; Ortiz, J. V.; Cioslowski, J.; Fox, D. J. Gaussian, Inc., Wallingford CT, 2009.

[2] Zhao, Y.; Truhlar, D. G. The M06 Suite of Density Functionals for Main Group Thermochemistry, Thermochemical Kinetics, Noncovalent Interactions, Excited States, and Transition Elements: Two New Functionals and Systematic Testing of Four M06-Class Functionals and 12 Other Functionals. *Theor. Chem. Acc.* **2008**, *120*, 215–241.

[3] Weigend, F.; Ahlrichs, R. Balanced basis sets of split valence, triple zeta valence and quadruple zeta valence quality for H to Rn: Design and assessment of accuracy. *Phys. Chem. Chem. Phys.* **2005**, *7*, 3297-3305.

[4] Zhao, Y.; Truhlar, D. G. Density Functionals with Broad Applicability in Chemistry. *Acc. Chem. Res.* **2008**, *41*, 157–167.

[5] (a) Miertuš, S.; Scrocco, E.; Tomasi, J. Electrostatic interaction of a solute with a continuum. A direct utilization of ab-initio molecular potentials for the prevision of solvent effects. *Chem. Phys.* **1981**, *55*, 117−129. (b) Pascual-Ahuir, J. L.; Silla, E.; Tuñón, I. GEPOL: An improved description of molecular surfaces. III. A new algorithm for the computation of a solvent-excluding surface. *J. Comput. Chem.* **1994**, *15*, 1127−1138. (c) Barone, V.; Cossi, M. Quantum Calculation of Molecular Energies and Energy Gradients in Solution by a Conductor Solvent Model. *J. Phys. Chem. A* **1998**, *102*, 1995−2001.

[6] Gonzalez, C.; Schlegel, H. B. Reaction path following in mass-weighted internal coordinates. *J. Phys. Chem.* **1990**, *94*, 5523−5527.

Cartesian coordinates (in Å) and total energies (in a.u., ZPVE included) of all the stationary points discussed in the text. All calculations have been performed at the PCM(CH_2Cl_2)-M06-2X/def2-SVP level.

INT0, E = -1108.047097

N	0.909276000	0.119985000	0.581176000
C	1.874745000	0.198935000	1.437804000
H	1.925633000	-0.660972000	2.116936000
C	0.662554000	-2.136125000	-0.446998000
C	2.042063000	-2.557196000	-0.029831000
C	3.162000000	-2.130140000	-0.616582000
H	4.145082000	-2.451918000	-0.267368000
H	0.674056000	-1.712193000	-1.458428000
H	-0.019120000	-3.000031000	-0.448292000
H	2.108172000	-3.231864000	0.831589000
C	0.064358000	-1.127627000	0.547572000
H	0.181558000	-1.539780000	1.559745000
H	3.129928000	-1.456836000	-1.479159000
C	2.889394000	1.257822000	1.639266000
H	3.677646000	1.123294000	0.880770000
H	2.491282000	2.268657000	1.509831000
H	3.329679000	1.129031000	2.632289000
C	-1.399440000	-0.781542000	0.375925000
C	-2.086246000	-0.255986000	1.477514000
C	-2.071706000	-0.950352000	-0.837347000
C	-3.421007000	0.124458000	1.361619000
C	-3.409571000	-0.571928000	-0.952044000
C	-4.082750000	-0.028246000	0.141889000
H	-1.568888000	-0.134995000	2.432908000
H	-1.551855000	-1.359427000	-1.703706000
H	-3.946050000	0.537065000	2.223839000
H	-3.926315000	-0.702281000	-1.903520000
H	-5.127630000	0.270030000	0.046653000
S	0.702707000	1.330584000	-0.772287000
O	0.311845000	0.525355000	-1.900193000
O	1.934399000	2.076975000	-0.781188000
C	-0.636283000	2.344743000	-0.227800000
H	-1.558476000	1.754190000	-0.242684000
H	-0.406796000	2.753737000	0.762372000
H	-0.664777000	3.145511000	-0.980284000

TS1, E = -1108.031693

N	-0.899414000	0.016645000	0.589066000
C	-2.153718000	0.454816000	0.994383000
H	-2.020583000	1.247686000	1.735797000
C	-0.516731000	2.064143000	-0.742407000
C	-1.916455000	2.327402000	-0.408492000
C	-2.887127000	1.346446000	-0.516157000
H	-3.915069000	1.588789000	-0.238480000
H	-0.419960000	1.503914000	-1.679355000
H	0.093921000	2.974116000	-0.752794000
H	-2.161695000	3.240620000	0.145185000
C	0.035484000	1.131177000	0.425277000

H	-0.040590000	1.707473000	1.357197000
H	-2.764956000	0.533969000	-1.238912000
C	-3.287775000	-0.454595000	1.417994000
H	-3.734126000	-1.021426000	0.600277000
H	-2.876411000	-1.161281000	2.154104000
H	-4.045468000	0.161402000	1.914225000
C	1.490152000	0.738524000	0.279176000
C	2.174174000	0.382197000	1.448839000
C	2.159966000	0.709087000	-0.945440000
C	3.503142000	-0.028776000	1.391020000
C	3.494339000	0.305111000	-1.001164000
C	4.164436000	-0.070400000	0.162239000
H	1.654084000	0.416650000	2.409075000
H	1.643149000	0.978117000	-1.866128000
H	4.025282000	-0.311113000	2.305947000
H	4.010028000	0.280658000	-1.961710000
H	5.206132000	-0.390479000	0.113269000
S	-0.760231000	-1.322485000	-0.521403000
O	-0.268356000	-0.784280000	-1.776227000
O	-2.042585000	-1.994969000	-0.499757000
C	0.454368000	-2.363125000	0.225541000
H	1.428059000	-1.862717000	0.209377000
H	0.118695000	-2.600003000	1.241168000
H	0.461114000	-3.261175000	-0.406229000

INT1, E = -1108.031645

N	0.903421000	-0.021704000	0.585964000
C	2.196042000	-0.503248000	0.940033000
H	2.037503000	-1.265838000	1.710814000
C	0.484445000	-2.062968000	-0.792675000
C	1.860029000	-2.337125000	-0.450766000
C	2.828617000	-1.311863000	-0.444052000
H	3.851278000	-1.610956000	-0.200252000
H	0.391482000	-1.451502000	-1.697688000
H	-0.152459000	-2.953249000	-0.812016000
H	2.103675000	-3.281021000	0.051148000
C	-0.033956000	-1.116861000	0.419564000
H	0.044001000	-1.718207000	1.334773000
H	2.759611000	-0.538551000	-1.217406000
C	3.292254000	0.430201000	1.437788000
H	3.763716000	1.007572000	0.640988000
H	2.847302000	1.121627000	2.166560000
H	4.043640000	-0.180759000	1.951367000
C	-1.490361000	-0.727192000	0.275610000
C	-2.170230000	-0.393385000	1.454422000
C	-2.163178000	-0.673120000	-0.946343000
C	-3.498554000	0.020799000	1.407609000
C	-3.496898000	-0.266137000	-0.990375000
C	-4.163316000	0.087441000	0.181893000
H	-1.647807000	-0.446695000	2.412319000
H	-1.650834000	-0.924369000	-1.874409000
H	-4.017456000	0.285998000	2.329431000
H	-4.014593000	-0.222329000	-1.949142000
H	-5.204758000	0.409516000	0.142012000
S	0.775789000	1.310709000	-0.517040000
O	0.273635000	0.794585000	-1.780081000

O	2.065172000	1.972143000	-0.499383000
C	-0.425705000	2.366116000	0.232216000
H	-1.404545000	1.875485000	0.223625000
H	-0.082910000	2.601084000	1.245797000
H	-0.429006000	3.263018000	-0.400873000

CH3-CHO, E = -153.586085

O	-1.228981000	-0.276405000	0.000000000
C	-0.231771000	0.397921000	-0.000004000
H	-0.307804000	1.511824000	-0.000004000
C	1.163566000	-0.148897000	-0.000011000
H	1.145089000	-1.244928000	-0.000469000
H	1.702095000	0.225499000	-0.883309000
H	1.701702000	0.224700000	0.883873000

INT2, E = -1261.639369

O	-1.433335000	2.482318000	-0.862675000
C	-2.627790000	2.300343000	-0.878935000
H	-3.030463000	1.274845000	-1.038806000
C	-0.354227000	0.725862000	1.657915000
C	-1.771275000	0.258843000	1.834351000
C	-2.798318000	1.072155000	2.077533000
H	-3.807256000	0.682465000	2.225996000
H	-0.311794000	1.824074000	1.689864000
H	0.257257000	0.345661000	2.494563000
H	-1.951796000	-0.822436000	1.793313000
C	0.294851000	0.296854000	0.337103000
H	-0.351216000	0.626354000	-0.483762000
H	-2.658328000	2.155307000	2.144337000
C	-3.641965000	3.384579000	-0.690224000
H	-3.148978000	4.348037000	-0.516073000
H	-4.285340000	3.437933000	-1.581226000
H	-4.294084000	3.126056000	0.157661000
C	1.685748000	0.841403000	0.093378000
C	2.132744000	0.945820000	-1.229479000
C	2.533396000	1.236815000	1.133199000
C	3.411817000	1.418616000	-1.509024000
C	3.815659000	1.711974000	0.852533000
C	4.257882000	1.799594000	-0.465820000
H	1.464325000	0.672861000	-2.049416000
H	2.201700000	1.191265000	2.171748000
H	3.746152000	1.500761000	-2.543963000
H	4.467208000	2.019393000	1.671345000
H	5.259204000	2.174300000	-0.682459000
N	0.305469000	-1.211771000	0.263546000
S	-1.083525000	-1.987421000	-0.633253000
O	-1.620747000	-2.963853000	0.282083000
O	-1.865054000	-0.867794000	-1.092353000
C	-0.252040000	-2.747318000	-1.991315000
H	0.433846000	-3.516896000	-1.623726000
H	-1.057096000	-3.197832000	-2.587806000
H	0.260338000	-1.961625000	-2.556367000
C	1.191825000	-1.911964000	0.881455000
H	1.968329000	-1.319086000	1.386375000

C	1.318737000	-3.377539000	0.979345000
H	0.472840000	-3.950519000	0.595656000
H	2.256590000	-3.655152000	0.469900000
H	1.477506000	-3.616220000	2.041565000

TS2, E = -1261.600293

O	2.162292000	2.034230000	-0.280930000
C	1.496562000	2.869081000	-0.855507000
H	0.447712000	2.632636000	-1.149972000
C	0.604244000	0.803389000	1.690010000
C	-0.431204000	1.892293000	1.705609000
C	-0.165614000	3.156981000	2.028695000
H	-0.951831000	3.913911000	2.036806000
H	1.576072000	1.183784000	2.047632000
H	0.331924000	-0.011022000	2.383302000
H	-1.453551000	1.604372000	1.439955000
C	0.848064000	0.204936000	0.350846000
H	0.329660000	0.616902000	-0.510637000
H	0.845715000	3.475050000	2.299178000
C	1.988852000	4.232776000	-1.198132000
H	3.029171000	4.362839000	-0.880870000
H	1.887550000	4.392069000	-2.281559000
H	1.338842000	4.972059000	-0.705936000
C	1.723470000	-0.900780000	0.116751000
C	1.859816000	-1.370218000	-1.207197000
C	2.447063000	-1.517644000	1.159312000
C	2.701902000	-2.436391000	-1.483617000
C	3.288793000	-2.583206000	0.875844000
C	3.413822000	-3.039351000	-0.441121000
H	1.293308000	-0.885859000	-2.005485000
H	2.353665000	-1.157118000	2.184511000
H	2.810613000	-2.802221000	-2.504382000
H	3.852963000	-3.062543000	1.675576000
H	4.077717000	-3.877777000	-0.657586000
N	-1.361403000	-0.874064000	0.265283000
S	-2.456907000	0.033812000	-0.684305000
O	-3.591130000	0.444318000	0.129548000
O	-1.624341000	1.062026000	-1.295105000
C	-2.973925000	-1.090088000	-1.952376000
H	-3.552121000	-1.907844000	-1.509388000
H	-3.600000000	-0.493122000	-2.627364000
H	-2.079442000	-1.452782000	-2.470198000
C	-1.736142000	-1.832578000	1.020110000
H	-0.916018000	-2.311058000	1.581087000
C	-3.087303000	-2.415263000	1.246664000
H	-3.902960000	-1.824764000	0.820800000
H	-3.087211000	-3.436176000	0.832717000
H	-3.236418000	-2.522100000	2.330264000

INT3, E = -540.410596

O	-1.062014000	0.834572000	-0.083270000
C	-2.147504000	1.125080000	-0.632202000
H	-2.440008000	0.574516000	-1.537597000
C	-0.925281000	-1.526898000	0.295205000
C	-2.376584000	-1.713535000	-0.029555000

C	-3.372691000	-1.269315000	0.744394000
H	-4.417631000	-1.403848000	0.457897000
H	-0.775899000	-1.330766000	1.367103000
H	-0.338725000	-2.418644000	0.030704000
H	-2.609950000	-2.205966000	-0.979939000
C	-0.311245000	-0.365761000	-0.499344000
H	-0.485473000	-0.489096000	-1.578245000
H	-3.172817000	-0.785338000	1.705727000
C	-2.960794000	2.230045000	-0.127929000
H	-2.585927000	2.607419000	0.828196000
H	-2.938686000	3.022304000	-0.896090000
H	-4.007293000	1.899203000	-0.061470000
C	1.141477000	-0.117748000	-0.214033000
C	1.549435000	0.326035000	1.048884000
C	2.091835000	-0.376914000	-1.203466000
C	2.903448000	0.511771000	1.314753000
C	3.448403000	-0.201513000	-0.929806000
C	3.853806000	0.243969000	0.327298000
H	0.807903000	0.536575000	1.822504000
H	1.771832000	-0.717936000	-2.190265000
H	3.219867000	0.863847000	2.297357000
H	4.188683000	-0.406311000	-1.704042000
H	4.914405000	0.386168000	0.539121000

MsN=CHMe, E = -721.201296

N	-0.453123000	1.142428000	0.001631000
C	-1.703383000	0.892773000	0.001161000
H	-2.350661000	1.783227000	0.001784000
C	-2.419450000	-0.417425000	-0.001427000
H	-1.754867000	-1.285825000	0.010504000
H	-3.087722000	-0.446811000	0.871855000
H	-3.064631000	-0.454485000	-0.891940000
S	0.623419000	-0.171992000	0.000807000
O	0.487767000	-0.907514000	-1.251474000
O	0.497103000	-0.900315000	1.258271000
C	2.159828000	0.700958000	-0.007228000
H	2.943976000	-0.065050000	-0.008078000
H	2.201859000	1.312031000	-0.914789000
H	2.208273000	1.316579000	0.896931000

TS3, E = -540.404527

O	1.096562000	0.663849000	0.361903000
C	2.368100000	0.505969000	0.664270000
H	2.585023000	-0.155491000	1.512458000
C	0.827064000	-1.463143000	-0.689790000
C	2.277371000	-1.553862000	-0.486187000
C	3.118171000	-0.544527000	-0.880099000
H	4.187609000	-0.611414000	-0.671762000
H	0.567462000	-1.007760000	-1.653858000
H	0.316184000	-2.425315000	-0.568156000
H	2.665490000	-2.336947000	0.174271000
C	0.287660000	-0.494292000	0.440014000
H	0.425318000	-0.974381000	1.420638000
H	2.800677000	0.160171000	-1.652619000

C	3.195076000	1.746879000	0.563886000
H	2.971381000	2.299286000	-0.355434000
H	2.919449000	2.368457000	1.430202000
H	4.263564000	1.519856000	0.632901000
C	-1.155384000	-0.138562000	0.219276000
C	-1.510008000	0.840179000	-0.714169000
C	-2.146765000	-0.827292000	0.920836000
C	-2.854131000	1.129707000	-0.937713000
C	-3.491766000	-0.541778000	0.687121000
C	-3.845831000	0.436821000	-0.240526000
H	-0.732782000	1.382841000	-1.255639000
H	-1.867738000	-1.587552000	1.653869000
H	-3.129461000	1.899335000	-1.659954000
H	-4.264183000	-1.081364000	1.236501000
H	-4.897833000	0.662881000	-0.420155000

INT4-Cl, E = -1000.706493

C	-1.632948000	1.654582000	-0.500204000
O	-0.286651000	1.382773000	-0.152859000
C	0.176917000	0.134508000	-0.612346000
C	-0.653725000	-1.001284000	0.003994000
C	-2.122871000	-0.781352000	-0.312223000
C	-2.560707000	0.607103000	0.118220000
H	-0.506732000	-0.988722000	1.095689000
H	-0.310782000	-1.973033000	-0.378588000
H	-3.601482000	0.795350000	-0.181643000
H	-2.499370000	0.680896000	1.216102000
H	-2.303973000	-0.920866000	-1.387183000
H	0.067715000	0.088436000	-1.715001000
H	-1.727788000	1.601412000	-1.603776000
Cl	-3.133792000	-2.039866000	0.503056000
C	-1.951708000	3.059435000	-0.033603000
H	-1.858650000	3.121252000	1.060184000
H	-1.253166000	3.775606000	-0.485848000
H	-2.976321000	3.335291000	-0.316775000
C	1.640637000	-0.009967000	-0.268179000
C	2.447862000	-0.878491000	-1.009568000
C	2.192887000	0.682319000	0.812791000
C	3.789145000	-1.056431000	-0.674542000
C	3.536371000	0.506787000	1.146008000
C	4.337052000	-0.363041000	0.405654000
H	2.022337000	-1.415427000	-1.861387000
H	1.562010000	1.365546000	1.381957000
H	4.410746000	-1.732698000	-1.263672000
H	3.961528000	1.054632000	1.988846000
H	5.388121000	-0.497148000	0.666001000
